# A comprehensive evaluation of advanced methods for identifying structural alerts using extensive toxicity data

**DOI:** 10.1186/s13321-026-01157-x

**Published:** 2026-01-30

**Authors:** Ning-Ning Wang, Yuan-Hang He, Xin-Liang Li, Shao-Hua Shi, You-Chao Deng, Shao Liu, Dong-Sheng Cao

**Affiliations:** 1 Department of Pharmacy, Xiangya Hospital, Central South University, Changsha, 410008 Hunan People’s Republic of China; 2 National Clinical Research Center for Geriatric Disorders, Xiangya Hospital, Central South University, Changsha, 410008 Hunan People’s Republic of China; 3 The Hunan Institute of Pharmacy Practice and Clinical Research, Changsha, 410008 Hunan People’s Republic of China; 4 Xiangya School of Pharmaceutical Sciences, Central South University, Changsha, 410013 Hunan People’s Republic of China; 5 School of Chinese Medicine, Hong Kong Baptist University, Hong Kong, People’s Republic of China

**Keywords:** Structural alerts, Comparison study, Bioalerts, PySmash, SARpy

## Abstract

With the disclosure of the important role of substructural alerts (SA) in drug development and toxicity evaluation, many automatic substructure extraction tools based on different theoretical knowledge have been reported in recent years. To compare the emphasis of various substructure extraction methods and the reliability of their results, we were encouraged to conduct a comprehensive analysis of seven representative tools to find the best one. In this paper, we introduced a well-designed evaluation of seven popular tools (Bioalerts, KRFP, MoSS, PySmash_circular, PySmash_group, PySmash_path, and SARpy) based on 43 toxicity datasets, consisting of four components: comparison of substructures derived by different methods, comparison of predictive models based on substructural rules, comparison of the efficiency of extracting toxic substructures, and the effect of SAs on quantitative structure–activity relationship (QSAR) predictive models. The results demonstrated that PySmash_circular performed best overall, with satisfactory results in substructure information carrying and the rule-based predictive models. PySmash_path and Bioalerts were also recommended for their similar performance to PySmash_circular, but the main problem was that they took too much time and generated too many substructures. Specifically, Bioalerts and PySmash_circular could obtain substructures carrying richer information, while SARpy had the best predictive rule-based models, but it only focuses on precision (PR) value in the evaluation of individual SA. More than that, the substructures obtained by all 7 methods can enhance the recognition ability of the QSAR models for toxic compounds and make them interpretable. Finally, we have also made a baseline substructure set of 43 toxicity endpoints available to the public to facilitate further development of drug research and environmental safety assessment in a rapid and accurate direction.

**Scientific Contribution**: Based on 43 toxicity datasets, we conducted a comprehensive evaluation of 7 representative substructure extraction tools from both the perspective of individual substructure and substructure-based models. This work not only enables users to make more autonomous choices of the optimal substructure extraction tool, but also provides the public with a benchmark substructure set of 43 toxicity endpoints, promoting the further development of computational toxicology.

**Graphical Abstract:**

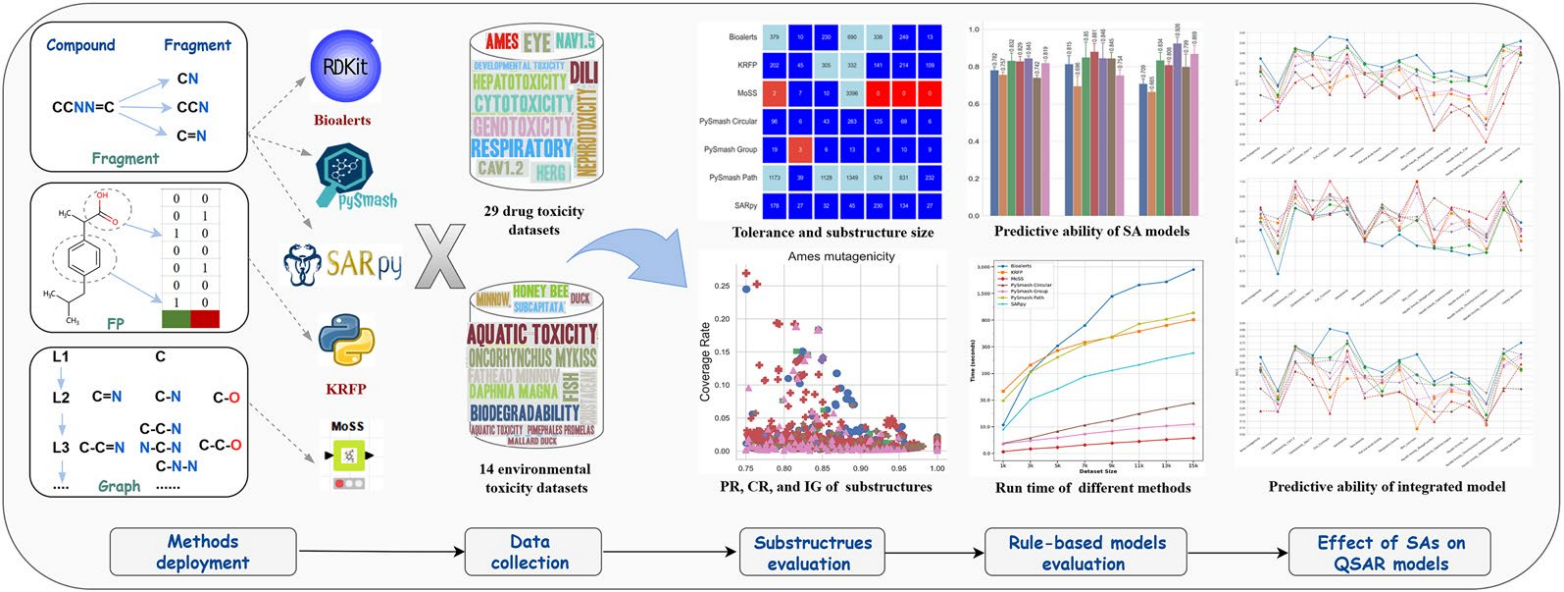

**Supplementary Information:**

The online version contains supplementary material available at 10.1186/s13321-026-01157-x.

## Introduction

The identification of potentially toxic compounds, which aims to assess the harmful effects of new compounds on humans, animals, plants, or the environment, is of great importance for drug discovery and environmental safety [1]. Usually, *in vitro* and* in vivo* tests are routinely performed to evaluate the toxic properties of all the new molecular entities, although they are time-consuming and laborious [2]. With the rapid development of computational science and the popularity of multidiscipline, *in silico* methods have shown great advantages in the field of toxicity assessment since they are green, fast, cheap, accurate, and most importantly they could be done before a compound is synthesized [3]. Among these methods, the most popular one is the construction of quantitative structure–activity relationship (QSAR) models, and many novel models with good performance are reported every year. Although these models have greatly contributed to the flourishing of computational toxicology, their inherent shortcomings, mainly interpretability and applicability domain, have limited their widespread application in regulatory agencies [4]. Another widely used method is structural Alert (SA). Firstly proposed by John Ashby in 1985, SA refers to a substructure or combination of substructures that play a key role in the toxicity of a compound [5]. It provides medicinal chemistry researchers with a concise, intuitive, and easily interpretable way to identify potentially toxic compounds and optimize their structures to reduce original toxicity [6].

Initially, the identification of toxic substructures mainly relied on expert systems, which were inferred from the experience or expertise of toxicologists, but with low accuracy. In recent years, the automatic recognition system of SA has been developed rapidly, which has better accuracy than the earlier expert system [7]. In general, the mining algorithms of SA can be roughly divided into three categories: fragment-based, graph-based, and fingerprint-based approaches. The fragment-based method aims to use cheminformatics tools to cut molecules into all possible fragments and calculate the frequency of occurrence of each fragment in toxic and nontoxic compounds to identify important substructures. For example, the CASE program proposed by Klopman reads and decodes the KLN code (a molecular linear coding routine) and then proceeds to generate the connectivity matrix and determines the type and number of occurrences of each possible subunit that exists in each molecule [8]. Bioalerts, a Python library for SA identification, relies on the RDKit implementation of the circular Morgan fingerprint algorithm to compute chemical substructures, which are derived by considering radial atom neighborhoods of increasing bond radius [9]. In 2013, Ferrari *et al.* introduced SARpy, which generates substructures of arbitrary complexity directly on the SMILES notation of structures, and the fragment candidates to become SAs are automatically selected based on their prediction performance on a training set [10]. More recently, Cao *et al.* developed a user-friendly and powerful tool, PySmash, to generate different types of representative substructures. It supports three substructure generation algorithms, self-designed functions, multiple output types, and the derived substructure application [11]. The graph-based mining algorithm treats chemical molecules as attributed graphs, in which each vertex represents an atom and each edge represents a bond between atoms. For example, GASTON, the GrAph Sequence True extraction, has achieved more efficient and flexible substructures search by first searching frequent paths, then frequent free trees, and finally cyclic graphs [12]. In addition, MoSS is also a popular tool based on a depth-first search in a tree of substructures, in which lists of embeddings into the set of molecules are processed and extended [13]. The fingerprint-based method is also a special fragment-based method that uses a predefined substructure library. For example, the Klekota-Roth fingerprint (KRFP) is a fingerprint encoding 4860 substructures proposed by Justin Klekota and Frederick P. Roth in 2008. They are generated by splitting the SMILES structure of each compound and are originally designed for molecular signatures for QSAR studies, but can also be used to derive structural alerts [14]. Overall, these automatic identification tools have preliminarily realized the identification of substructures with low cost and high efficiency, and have important practical significance in different stages of drug development and environmental safety evaluation.

In the current context where various tools are flourishing, to assist drug researchers in finding an SA identification tool that is easy to use and yields reliable results, we were encouraged to conduct comparative assessments of representative tools in different categories to determine their unique characteristics. In this paper, we provided an exhaustive analysis of seven popular SA identification methods, namely Bioalerts (fragment-based), KRFP (fingerprint-based) and MoSS (graph-based), PySmash (three, fragment-based), SARpy (fragment-based), using extensive toxicity data consisting of 43 datasets. The whole evaluation process consists of four stages: Firstly, based on the obtained substructures, a preliminary evaluation of different methods was carried out, including the comparison of the tolerance on different data, the size of their derived rule sets, the information carried by the individual substructures, and the comparison with ToxAlerts substructures. Secondly, the overall prediction ability of the model composed of the derived substructures was evaluated to further confirm the practicability of the substructures. Thirdly, the extraction efficiency of 7 methods for toxicity substructures was uniformly compared, and fourthly, the specific effect of substructures on QSAR models was comprehensively analyzed. Finally, the substructures obtained in this study were screened reasonably to form a benchmark substructure set, which will be freely provided to the public. Based on the above evaluation and comparison, we hope to not only provide the community with informative substructures of some important toxicity endpoints, but more importantly, help pharmaceutical chemists more comprehensively understand the different SA identification tools and be able to choose the most appropriate one according to their needs, thus accelerating the process of drug development and environmental safety evaluation.

## Materials and methods

### Data collection

To comprehensively evaluate the usefulness and reliability of the above seven SA identification tools, we searched and collected as many toxicity datasets as possible from the recently published literature [15–23]. In fact, we collected a total of 43 toxicity datasets from 9 literature sources. Among them, 26 datasets were collected from admetLab 3.0, 6 from admetSAR 3.0, 3 from AquaticTox, and 6 from other sources. All the original data were binary classification labels, and a single high-quality data source ensured that no compound had multiple values. For these datasets, we selected drug- and environment-related endpoints and pretreated them with the following steps to ensure their quality: (1) checking and washing all the molecules by molecular operating environment (MOE, version 2022) to remove solvent or saline ions [24]. (2) converting them to canonical SMILES using OpenBabel [25]. (3) removing duplicate compounds. Finally, a high-quality toxicity database consisting of 29 drug-related endpoints and 14 environment-related endpoints was obtained. All the toxicity endpoints and their datasets can be seen in the Supporting Information_1.

### Representative tools for SA identification

In this study, we focused on evaluating seven popular approaches: Bioalerts, KRFP, MoSS, PySmash (circular, group and path) and SARpy. The input format of the compounds for all methods was canonical SMILES, and the output substructures were uniformly converted to SMARTS for further comparative study. A brief description of each method and the parameters setting are shown below.

1. Bioalerts (https://github.com/isidroc/bioalerts) is a python library that can automatically identify SAs from categorical or continuous bioactivity data sets. It relies on the circular Morgan fingerprint algorithm implemented by RDKit to compute substructures that are obtained by considering radial atom neighborhoods of increasing bond radius [9]. For the derivation of structural alerts from categorial bioactivity data sets, all compounds are classified into a training set and a test set. The training set is used to derive the substructure dictionary, which is employed to calculate the P-values of the substructures present in the molecules from the test or external sets. Through the calculation and screening of three parameters (threshold_nb_substructures, threshold_pvalue, and threshold_frequency), the qualified substructure will be finally obtained. In this study, the parameters were set as follows: minHits = 5, threshold frequency = 0.75, Bonferroni = True.
2. KRFP is a predefined non-redundant substructure fingerprint library published by Justin Klekota and Frederick P. Roth in 2008 (The original code is Available at https://academic.oup.com/bioinformatics/article/24/21/2518/192573?login=false#394591319). It contains 4860 substructure fingerprints and can be used to characterize molecules and detect structural alerts [14]. The substructure mining process based on KRFP consists of three steps: using PaDEL-Descriptor (http://yapcwsoft.com/dd/padeldescriptor/, V2.20) to calculate fingerprints, calculating the positive values of each fingerprint, and filtering out the important substructures based on the positive rate and minimum hit [26]. In KRFP, the parameters were set as follows: f-scores ≥ 0.005, positive ≥ 0.75.
3. MoSS (https://github.com/isidroc/bioalerts) is an efficient graph substructure mining algorithm for finding closed frequent fragments of molecules. This algorithm is based on a depth-first search of the substructure tree, and processes and expands the list embedded in the molecular set. The algorithm searches as follow: The given core structure is embedded into all molecules, resulting in a list of embeddings. In a second step, each embedding is extended in every possible way. And then, the extended embeddings are sorted into equivalence classes, each of which represents a new substructure. Finally, advanced pruning methods are applied to obtain the most relevant substructures. Its core advantages are a structural pruning scheme, which tries to minimize the number of times a fragment is considered in the search, and two advanced pruning techniques, which speed up the search considerably [13]. In this study, MoSS was implemented in python with the following parameters: the minimum focus support = 10%, the maximum complement support = 5%. The other parameters were set as default.
4. PySmash (https://github.com/kotori-y/pySmash) is a user-friendly and integrated tool for the automatic generation of representative substructures. This tool provides users with three types of substructure generation algorithms, including circular-based, path-based, and functional group-based algorithms. In the circular-based substructure generation algorithm, the atoms are assigned with the integer identifiers and they are collected into an initial set. As the radius expands, this set includes all the identifiers found in both the previous iterations and the current one. The path-based substructure generation algorithm is proposed to search all the patterns in a molecule within a particular restriction of path-length. As for the functional group-based substructure generation algorithm, it is intended to identify functional groups based on heteroatoms and aromatic atoms. In PySmash, after loading a file that includes SMILES and toxicity labels, we can choose the desired method of generating structural fragments. There are two types of parameters available for adjustment: fragment parameters and running parameters [11]. The fragment parameters of different methods were set as follows: for the circular-based method, mini radius = 1, max radius = 4; for the path-based method, mini path = 1, max path = 7; for the group-based method, no parameter needs to be adjusted. The running parameters for the three methods were uniformly set to minNum = 5, minACC = 0.75, p-value = 0.05, Bonferroni = True.
5. SARpy (https://sourceforge.net/projects/sarpy/) is a system focusing on the important structural features hidden in the dataset. It differs from other approaches in that it can extract relevant knowledge in the form of SA during the learning phase and ultimately produce a small set of effective rules [10]. Given a training set of molecular structures, with their experimental activity binary label, the whole extraction process is done in three steps starting just from the structural SMILES: a new recursive algorithm is used to obtain all possible substructures, each of which is verified in the training set as a potential SA, and a simplified rule set is extracted from the large pool of substructures. In our SA extraction process, the parameters were set to atoms number = 2 ~ 15, minHits = 5 and the precision is set to OPTIMAL (minprecision = 0.5).

**Fig. 1:**
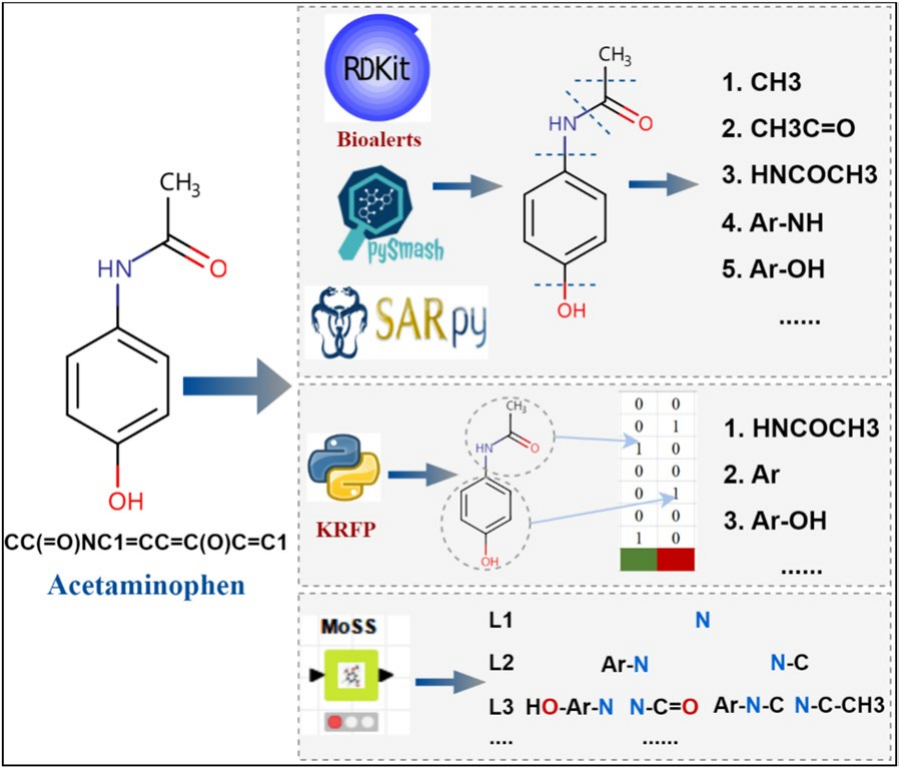
A schematic diagram illustrating the process of generating substructures using each tool

To present the process and results of each tool generating the substructures more intuitively, we take the compound acetaminophen as an example to illustrate different methods, as shown in Fig. 1. Additionally, in order to ensure the rationality and reliability of this comparative study, considering that the constraints for generating substructures of MoSS and SARpy are different from those of other methods, we will screen their substructures by [minHits ≥ 5 and minprecision ≥ 0.75] and then conduct subsequent comparative studies with other methods.

### Evaluation of substructures obtained from different tools

#### Assessment of individual substructures obtained from seven methods

To evaluate the reliability and practicability of SAs derived by different methods, for each substructure, we calculated three commonly used indicators that have been reported in the literature in this field, namely precision (PR), coverage rate (CR) and information gain (IG), respectively. These statistical parameters are defined as follows:

1 $${\mathrm{PR}}\, = \,\frac{{N_{sub\, - \,positive} }}{{N_{sub} }}$$

2 $${\mathrm{CR}}\, = \,\frac{{N_{sub} }}{{N_{total} }}$$

where *N*_*total*_ means the total number of compounds in a toxicity dataset, *N*_*sub*_ means the number of all compounds that contain the substructure, *N*_*sub-positive*_ means the number of positive compounds that contain a certain substructure [7].

To find substructures with both a high coverage rate and high precision, we can use a comprehensive indicator, IG, which is based on entropy theory and has been widely applied in statistics and data-mining methods. IG is defined as the difference between the information entropy of the origin data set and the weighted average information entropies of the two data sets separated by a substructure. And the information entropy (E(P)) is a value used to describe the degree of information disorder or certainty. The higher the degree of disorder, the greater the entropy value, and the more difficult it is to be predicted; the lower the degree of disorder, the smaller the entropy value, and the easier it is to be predicted. For an ideal toxic substructure X, it can reliably divide the disordered original data into a positive set and a negative set with lower information entropy. At this point, the substructure X has a higher IG value. The calculation formulas of information entropy (E(P)) and IG are shown below [27]:

3 $${\mathrm{E}}\,\left( {\mathrm{p}} \right)\, = \, - p\, \times \,\log \,p\, - \,\left( {1\, - \,p} \right)\, \times \,\log \left( {1\, - \,p} \right)$$

4 $${\mathrm{IG}}\, = \,E\,\left( {P\,\left( {X\, = \,1} \right)} \right)\, - \,P\,\left( t \right)\, \times \,E\,\left( {P\,\left( {X\, = \,\left. 1 \right|t} \right)} \right)\, - \,P\,\left( {\overline{t}} \right)\, \times \,E\left( {P\,\left( {X\, = \,\left. 0 \right|\overline{t}} \right)} \right)$$

for a compound in a specific dataset, it can be categorized positive (X = 1) or negative (X = 0) based on a binary property X. *p* means the probability of molecules in one category and t is defined as compounds that contain the substructure and used t̅ means compounds that do not contain the substructure. P(X = 1) means the probability of a molecule in positive group. The open-source code for IG calculations is derived from [28].

#### Evaluation of predictive ability of models composed of derived substructures

To further evaluate the ability of each method to identify SA, we treated a set of substructures based on each method and each data set as a predictive model and evaluated its predictive power on both internal and external datasets. For each data set, we randomly divided them into a training set and a test set in a ratio of 8:2, where the training set was used for substructure extraction and the test set was used for external validation of the overall model of the substructures. The process was repeated five times to ensure the stability of the predicted results. The predictive power of these SA-based models was measured by three statistical parameters: accuracy (ACC), positive predictive value (PPV), and Matthews’s correlation coefficient (MCC). These indicators are defined as follows:

5 $${\mathrm{ACC}}\, = \,\frac{TP\, + \,TN}{{TP\, + \,FN\, + \,TN\, + \,FP}}$$

6 $${\mathrm{PPV}}\, = \,\frac{TP}{{TP\, + \,FP}}$$

7 $$MCC\, = \,\frac{TP\, \times \,TN\, - \,FP\, \times FN}{{\sqrt {\left( {TP\, + \,FP} \right)\, \times \,\left( {TP\, + \,FN} \right)\, \times \,\left( {TN\, + \,FP} \right)\, \times \,\left( {TN\, + \,FN} \right)} }}$$

where TP, FP, TN and FN represent the number of true positives, false positives, true negatives and false negatives, respectively.

## Results and discussion

### Individual substructures derived from seven methods

#### Tolerance of different methods for different toxicity datasets

**Fig. 2:**
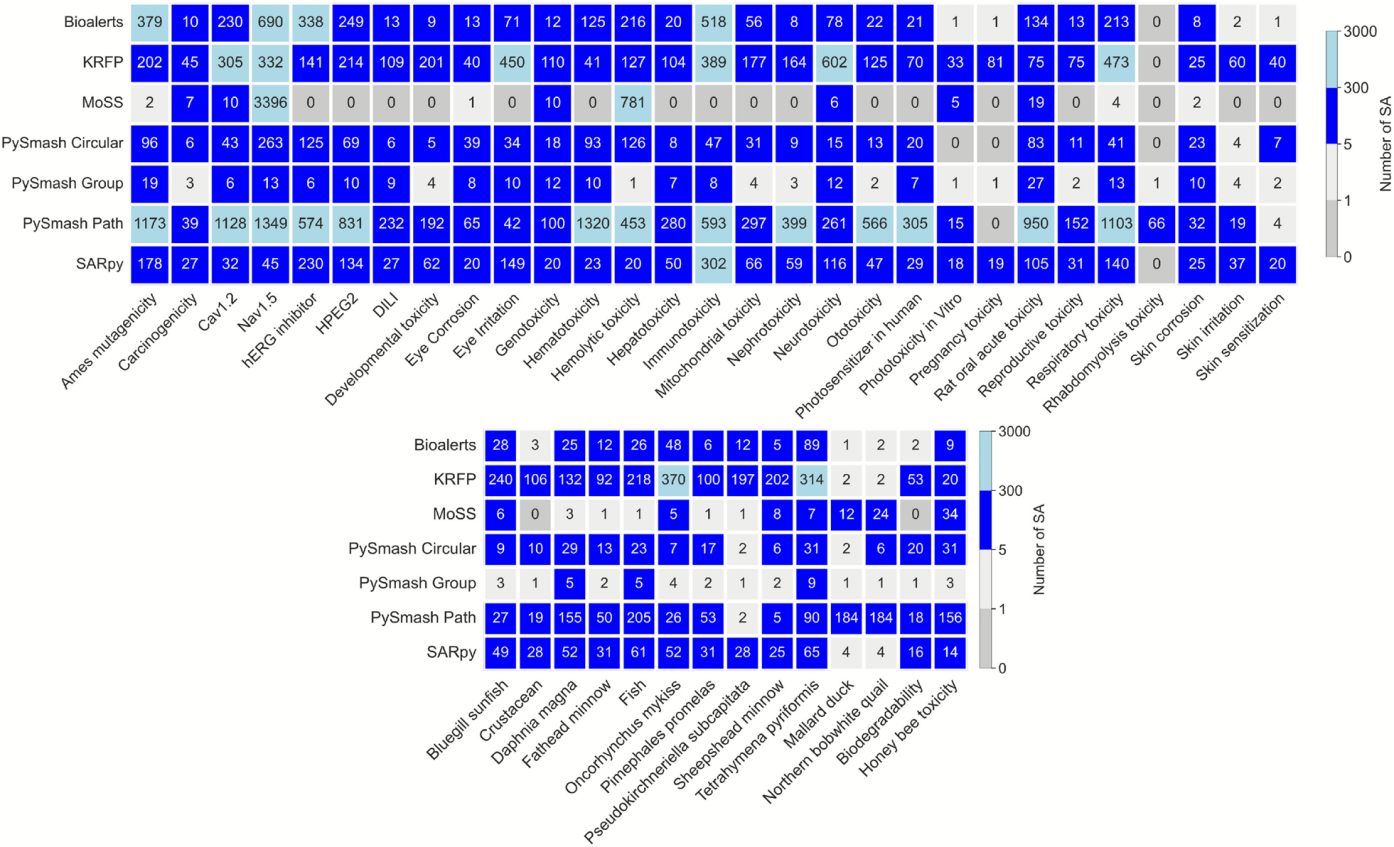
The number of substructures generated by seven different methods

After extracting informative substructures from 43 toxicity datasets using seven methods, the heat map was applied to visualize the number of substructures generated by different methods, as shown in Fig. 2. As can be seen from this figure, there are cases where substructures cannot be extracted when a certain method is applied to some data sets. In other words, different substructure extraction methods have different tolerances for different data sets. In this part, the “Tolerance” refers to the ability of a certain substructural extraction tool to successfully extract the SAs from the different toxicity datasets provided as input under the current parameters. If a tool can successfully extract SAs from a larger number of datasets, then we consider that it has a higher tolerance for different toxicity data; conversely, the opposite is also true. To further analyze the tolerance of the seven methods for 43 toxicity datasets, we sorted out and summarized the items that failed to obtain substructure in the extraction process. The results showed that PySmash_group has better data tolerance than the other six methods and can successfully find meaningful substructures for all 43 toxicity datasets. MoSS has the worst tolerance for different datasets, failing to extract substructures 19 times out of 43 datasets, followed by the PySmash_circular method with 3 failures (phototoxicity_in vitro, pregnancy toxicity and rhabdomyolysis toxicity). The substructure extraction of Bioalerts, KRFP, PySmash-path and SARpy on 43 toxicity data all experienced only one failure, and only PySmash_path was on pregnancy toxicity dataset, while the other three were on rhabdomyolysis toxicity dataset. In fact, five of the seven methods failed to successfully extract substructures from rhabdomyolysis toxicity data. This is because the data set is very unbalanced, with positive data accounting for only about one-tenth of the total data, and it is really difficult to find an eligible substructure when the precision required is greater than 0.75. In general, PySmash_group, Bioalerts, KRFP, PySmash-path, and SARpy may be more tolerant of different data than PySmash_circular and MoSS when using default parameters. Considering that different parameters can have a profound impact on the extraction results, appropriate adjustments to extraction parameters, such as Bonferroni correction in PySmash and Minimum focus support and Maximum complement support in MoSS, may improve the tolerance of PySmash and MoSS to specific datasets and thus help us obtain satisfactory and informative substructures.

#### The size of substructure set

In the course of this comparative study, we found that the number of substructures obtained by different methods was quite different, ranging from a few to hundreds or even thousands. In the practical work of toxicity research, we hope to fully and accurately explain the existence of toxicity with relatively few substructures. Therefore, in addition to the information carried by an individual toxic substructure, the size of the substructural set is another important indicator to be considered in comparative studies. According to Fig. 2, we can see that PySmash_group generated the fewest substructures among all methods and the number of substructures obtained from 43 toxicity datasets was less than 30, and most of them were in single digits. The main reason is that PySmash_group aims to identify only functional groups based on heteroatoms and aromatic atoms, which are destined to produce relatively fewer substructures than other methods [29]. This was followed by MoSS, which extracted substructures on only 24 toxicity datasets, but 18 of them had single-digit numbers. Unusually, MoSS identified more than 3000 substructures from the Cardiotoxicity_Nav1.5 dataset, which is too many for itself or even for all seven methods. After analyzing the original compounds, we guessed that this could be due to the large molecular weights and extremely similar structures of most of the compounds in the dataset. In contrast, the method that produced the most substructures is PySmash_path, with 13 of the 43 toxicity datasets having more than 300 substructures, 11 having between 100 and 300 substructures, and even 5 having more than 1,000 substructures. The path-based substructure generation algorithm can search all patterns in a molecule under a specific path length limit, which is not only time-consuming but also possible to identify some redundant substructures, which also leads to an increase in the number of substructures [30]. PySmash_circular and SARpy also performed well in terms of the number of substructures, with the vast majority of substructure sets smaller than 100 and no substructure sets larger than 400. Bioalerts and KRFP fared slightly worse, with KRFP, in particular, having eight substructure sets larger than 300 in size, possibly related to excess substructure information. In summary, except PySmash_path, the other six SA extraction methods can obtain the appropriate number of substructures on different toxicity datasets, especially PySmash_group, PySmash_circular, and SARpy, whose substructures of all toxic endpoints were less than 500.

#### Comparison of individual substructures

**Fig. 3:**
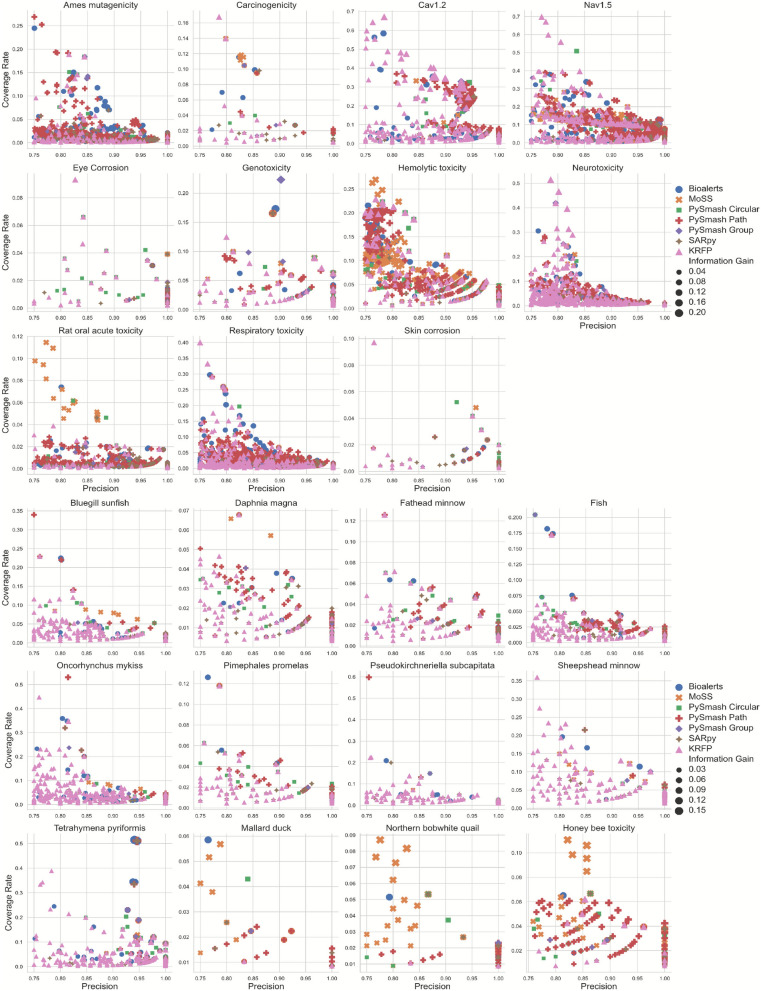
The distribution of PR and CR values for the substructures of 23 toxicity endpoints

After evaluating the data tolerance and the size of substructures set of seven different methods, we will further analyze the differences in the carrying information of substructures obtained by these methods in detail in this section. As mentioned in Sect. 3.1.1 earlier, among the 43 toxicity data sets, 20 could not be extracted with substructures using all 7 methods simultaneously. Therefore, in this section, we will only conduct the subsequent discussion based on the remaining 23 data sets. That is, in this part, a total of 161 substructure sets were obtained through the 7 methods (23 × 7). To quantitatively measure the information capacity of each substructure, we introduced three important indicators, PR, CR, and IG, which can evaluate the ability of the substructure to predict the toxicity of the compound. For each substructure in the SA set, we calculated its PR, CR and IG values respectively and made them into individual scatter plots based on toxicity endpoint. Figure 3 shows the distribution of PR and CR values for the substructures of 23 toxicity endpoints, where the size of the dots represents the IG value and the color of the dots represents different methods. As can be seen from this figure, for all the substructures of 23 toxicity endpoints, the value of PR is between 0.750 and 1.000, mainly distributed between 0.800 and 0.950, while the value of CR is between 0.001 and 0.700, mainly distributed between 0.001 and 0.350. For most toxicity endpoints, there appears to be a similar negative correlation between PR and CR values for their substructures, that is, with the increase of PR value, CR value has a tendency to decrease. Theoretically, a qualified structural alert should have both excellent coverage rate and precision, ideally with PR and CR values as close to 1 as possible, but this is almost impossible to achieve in practice. Therefore, substructures with the largest IG values usually have relatively moderate PR and CR values, distributed in the middle region of the entire graph.

**Fig. 4:**
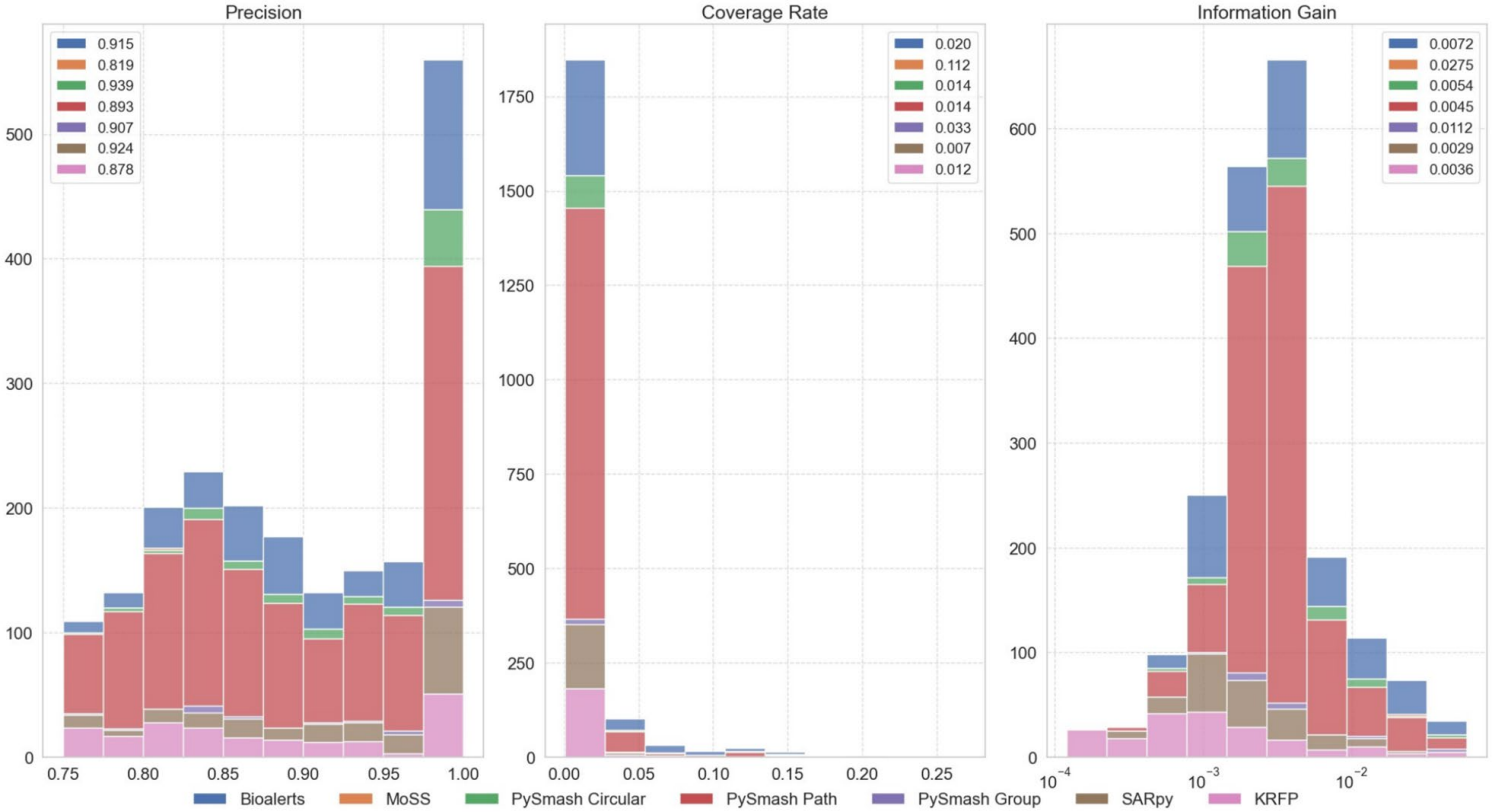
The distribution of PR, CR and IG values of Ames substructure obtained by 7 methods

To more intuitively show the differences between the substructures obtained by different methods, we plotted the distribution of PR, CR and IG values of the substructures for each toxicity dataset. The substructure distribution figures of 23 toxicity datasets can be seen in the Supporting Information_2. Here, we take Ames mutagenesis as an example to analyze the substructural characteristics derived from each method. Figure 4 shows the distribution of PR, CR and IG values of Ames substructure obtained by 7 substructure extraction methods, respectively. As can be seen from the figure, MoSS obtained the best CR and IG mean values of 0.112 and 0.027 respectively, while its PR mean was the lowest among all methods, only 0.819. This phenomenon was also observed on the other 22 datasets, where MoSS had the highest CR and IG averages on 16 and 15 datasets, respectively, but the lowest PR averages on 11 datasets. Although the average values of PR, CR and IG of the Ames substructure obtained by Bioalerts were not the highest, they all ranked in the top three, and their overall substructure detection ability was the strongest. In fact, PySmash, especially PySmash_circular, behaved similarly to Bioalerts on PR, CR, and IG values across all 23 datasets, which makes sense because they all fragment compounds based on Morgan fingerprints. However, the average CR value of PySmash_path-based substructures on the Pseudokirchneriella subcapitata data was much higher than that of the other six methods. To delve into the reason for this, we compared the number of substructures and CR values of different methods on different datasets. We discovered that for other toxic datasets, the PySmash_path would always obtain the largest number of substructures, although there were several substructures with higher CR values, the overall CR mean was very low. The anomaly on the Pseudokirchneriella subcapitata might be due to the fact that the number of substructures obtained on this dataset was relatively small, only 2, which resulted in its CR mean being higher than that obtained by other methods. The Ames substructures identified by SARpy had high mean PR, but most of their CR values were distributed between 0 and 0.025, which resulted in the lowest mean IG. SARpy also performed worst in the CR values of the other 22 data sets, with 10 having the lowest mean values. The Ames substructures detected by KRFP had the second-lowest mean PR, CR, and IG, and it also performed the worst in CR and IG values when applied to other datasets. In summary, the results of seven methods applied to different toxicity datasets demonstrated that Bioalerts and PySmash performed satisfactorily overall, SARpy focused more on PR values, MoSS focused more on CR values, and KRFP performed poorly in all aspects. Based on the 301 substructures extracted by seven tools, we aim to eventually provide toxicologists with a structure alert benchmark database covering 43 toxicity endpoints. Therefore, we conducted a consensus analysis on the substructure sets obtained from the 7 methods in this section. That is, for a specific toxicity endpoint, we identified the toxic substructures that appeared simultaneously in all 7 substructure sets, and used them as the benchmark substructure set for this toxicity endpoint, making it available for public access and reference. During the execution process, in order to find the appropriate number of shared substructures for each toxic endpoint, we set some prerequisites: (1) For each substructure, remove the position marking information from the original SMARTS structure and convert it into a standardized SMILES. (2) If there is an inclusion relationship between the two compared substructures, they are considered to be the same substructure, and the simpler substructure is taken as the final substructure. (3) When searching for the common substructures for various toxicity endpoints, exclude methods that generate fewer than 20 substructures, and include the toxic substructures that appear in all remaining methods. During this process, we further evaluated the degree of overlap in the substructures produced by different methods for each toxicity endpoint. Specifically, for a toxicity endpoint, we counted the number of identical substructures obtained from any two of the seven methods. The original substructure sets, the benchmark substructure sets, and the results of overlap degree of seven methods can be seen in the Supporting Information_3.

#### Compared with substructures in ToxAlerts

To further evaluate the reliability of the substructures detected by different methods, we intended to compare our toxic substructures to the structural alerts reported in previous publications. ToxAlerts is a web-server of structural alerts, which collected SAs defined by experts or detected by computational tools. It enables users to search structural alerts, introduce your own alerts and screen chemical libraries for alert-hitting compounds, and has been cited by several studies in the field of toxicity [31–33]. Therefore, we selected the toxic substructures within ToxAlerts as the reference for the comparative analysis. In this part, we collected two substructure sets from ToxAlerts identical to our toxicity endpoints, namely oral acute toxicity and Aquatic toxicity_fish. The top 10 substructures ranked by IG values extracted by seven methods were compared with ToxAlerts substructures respectively, and their results can be seen in the Supporting Information_4. For Aquatic toxicity_fish, most of the substructures identified by Bioalerts, KRFP and PySmash_path overlapped with those listed in ToxAlerts, followed by PySmash_circular and SARpy. MoSS detected only one substructure, which was also in the ToxAlerts library. Most of the top ten substructures obtained by Bioalerts were very similar, which illustrated the universality and redundancy of this method. As for oral acute toxicity, PySmash_group detected the most identical substructures with ToxAlerts, followed by PySmash_circular and SARpy, and several more general substructures were identified. The remaining substructures were all imidazole and trifluoride compounds, which were not present in ToxAlerts, but the results of this study suggested that they may be important for oral acute toxicity. In summary, Bioalerts, KRFP, and PySmash_path appear to perform better from a ToxAlerts perspective, PySmash_circular, SARpy, and PySmash_group slightly less well, and the substructures identified by MoSS were more general than those identified by other methods.

### Evaluation of overall predictive models based on different methods

**Fig. 5:**
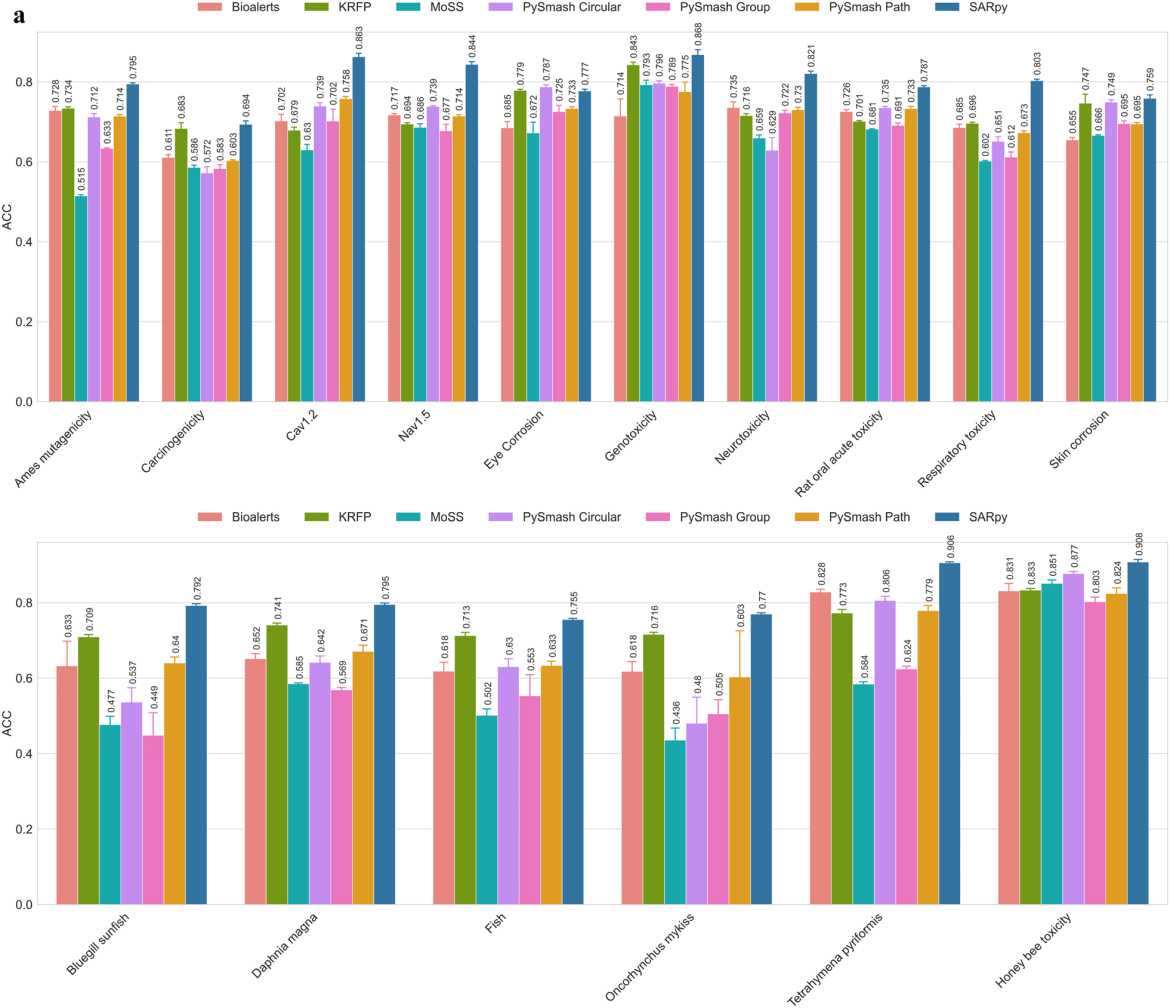

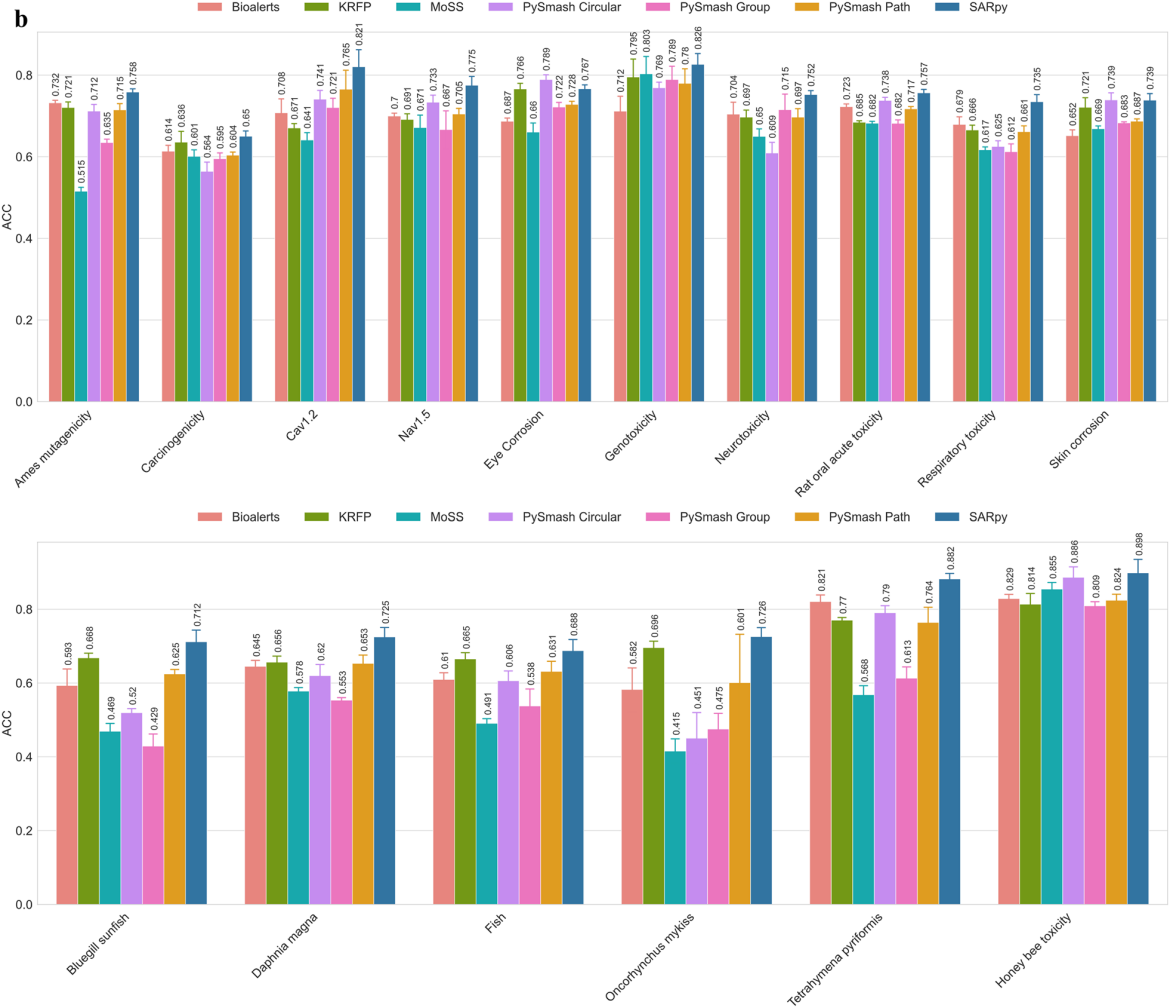
The ACC values of the prediction models on the training sets (**a**) and test sets (**b**)

To further evaluate the practicability of the substructures extracted by each method, we took the substructure set obtained by each method in the previous part as a prediction model and evaluated its prediction ability on the training set and the external test set respectively. In the substructure extraction process, there were a total of 27 toxicity datasets that could not be successfully applied to each substructure identification method. Therefore, they were excluded from this part of the comparison. In this section, we obtained a total of 560 prediction models covering 16 toxicity endpoints and 7 substructure extraction methods. To evaluate these models as impartially as possible, three statistical parameters with different emphases were cautiously chosen, namely, ACC, which measures the accuracy of the model's overall prediction, PPV, which focuses on the positive prediction result, and MCC, a comprehensive indicator that considers the relationship between true positives, true negatives, false positives, and false negatives [34]. For each toxicity endpoint, we calculated the average of its five random results for each method and used that value as the final metric for comparison to reduce random error. The ACC values of the prediction models based on 7 different substructure extraction methods and 16 toxicity datasets are shown in Fig. 5 and the corresponding figures of PPV and MCC values can been seen in the Supporting Information_5.

According to Fig. 5 and Supporting Information_5, we can reach the following consensus through comprehensive sorting and analysis: first of all, from the perspective of ACC value, both internal and external validation results showed that SA models derived from SARpy have the best overall prediction ability. Specifically, the optimal models for 15 of the 16 toxicity endpoints were SARpy-based, with ACC values ranging from 0.694 to 0.908 in the training set and 0.650 to 0.898 in the test set. The only remaining toxicity endpoint is Eye_Corrosion, whose optimal model was derived from PySmash_circular. KRFP ranked second in this competition, with 16 toxicity predictive models with ACC values ranging from 0.679 to 0.843 in the training set and 0.636 to 0.814 in the test set. As for the other five methods, MoSS performed worst in ACC (ACC_tr: 0.436 ~ 0.851, ACC_te: 0.415 ~ 0.855), followed by PySmash_group (ACC_tr: 0.449 ~ 0.803, ACC_te: 0.429 ~ 0.809), while Bioalerts, PySmash_path, and PySmash_circular all performed similarly with commendable ACC values. Secondly, from the point of view of accurately identifying positive compounds (i.e., PPV values), SARpy remained the best, with the highest PPV values in 7 of the 16 endpoints, ranging from 0.849 to 0.941 in the training set and 0.754 to 0.946 in the test set. The worst performer was KRFP, which had the worst PPV value for 13 endpoints, ranging from 0.672 to 0.819 in the training set and 0.641 to 0.783 in the test set. For the three methods deployed in PySmash, they performed only slightly worse than SARpy on PPV values, and PySmash_group performed better than PySmash_circular and PySmash_path. The two remaining methods, Bioalerts and MoSS, also performed well, with similar results as PySmash_path and PySmash_circular, respectively. Finally, in terms of the MCC values of the 560 substructural models, the performance of each method was similar to the results of the ACC values above, and these models based on SARpy still have the highest average MCC values (MCC_tr: 0.430–0.746, MCC_te: 0.327–0.717). KRFP behaved similarly to PySmash_circular, but its MCC value fluctuates greatly (MCC_tr: 0.147 ~ 0.681, MCC_te: 0.081 ~ 0.588), which seems to imply that its predictive power is less stable than PySmash_circular (MCC_tr: 0.430 ~ 0.746, MCC_te: 0.152 ~ 0.682). The predictive power of rule models obtained by other methods was ranked by MCC values as follows: PySmash_path, Bioalerts, PySmash_group and MoSS.

In summary, from the ACC, PPV and MCC information of the above substructural model, we can draw the following conclusions: (1) SARpy-based substructure models performed best in terms of whole prediction accuracy and the balance of prediction ability for positive and negative compounds, which should be attributed to the underlying logic of original substructure extraction in the SARpy method. SARpy introduces a new recursive algorithm that takes into account all combinations of key breaks that directly act on SMILES strings. The selection of substructures is based on the order of likelihood ratio, which takes into account the prediction accuracy of both positive and negative compounds. This is why SARpy-based substructure models have better MCC values than other models, which is also consistent with the original developers’ argument [10]. Another feature that makes SARpy better than most similar data mining systems is that it generates a smaller set of rules that can be used to make expert predictions and can also be read by human experts to uncover new clues in the field of toxicity research, which we have also discussed earlier. (2) Overall, PySmash is second only to SARpy in its ability to extract substructures, and even slightly better in terms of predicting positive compounds for some toxicity endpoints. This is because, unlike SARpy, the primary metric for evaluating substructures in Pysmash is PR [11]. That is, PySmash pays more attention to the prediction accuracy of positive compounds, which directly leads to the better performance of PySmash-based models on PPV values, but slightly worse than SARpy on ACC and MCC values. Of the three methods provided by PySmash, the rule models based on PySmash_circular and PySmash_path have similar predictive ability, while the models based on PySmash_group performed the worst. This may be because the functional group-based substructure generation algorithm is mainly based on heteroatoms and aromatic atoms to identify functional groups, and its ability to obtain rich structural information is not as good as the other two methods. In addition, considering that PySmash_path takes too much time to generate substructures and produces several or even tens of times as many substructures as the other two methods, we believe that PySmash_circular is the best and most practical of the three methods. Bioalerts, also a fragment-based approach, performed almost as well as PySmash in every respect. Overall, the substructures identified by the five fragment-based methods have high accuracy and interpretability, which may benefit from the fact that unlike fingerprint- and graph-based methods, they can smash the chemical compounds to get all the substructures to find more meaningful toxic fragments. (3) KRFP, as the fingerprint-based substructure generation method, also performed well overall in this evaluation. In particular, it was second only to SARpy in ACC and MCC values but ranked last in PPV values. (4) The overall performance of MoSS was poor compared to other methods, and the predictive power of the models was only acceptable when predicting positive compounds. In addition to the limitations of the method itself, part of the reason is that the threshold set by the default parameter is too narrow to detect enough substructures, resulting in more false negative results and low ACC and MCC values. In conclusion, the substructure models based on fragment-based approaches, such as SARpy, PySmash and Bioalerts, performed well in all aspects of prediction ability, while the positive recognition rate of the KRFP model was insufficient, and the overall prediction ability of the MoSS model was the worst among the seven methods.

### Comparison of the efficiency of different methods

**Fig. 6:**
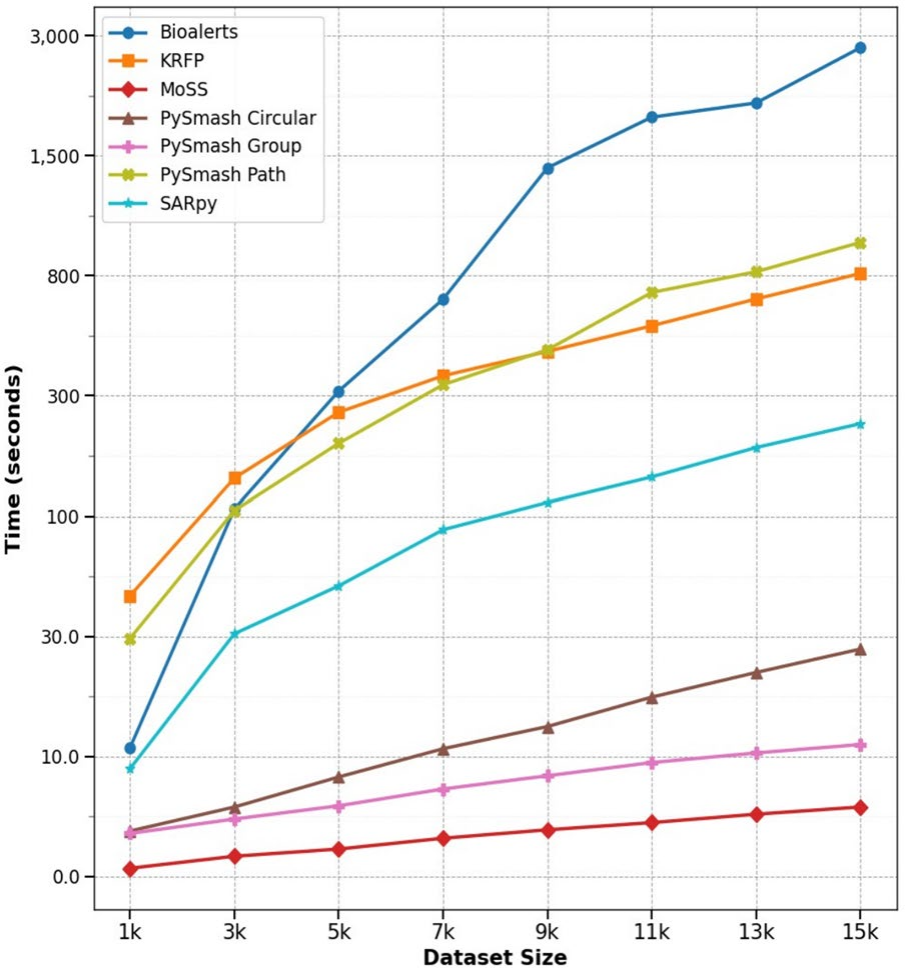
Time spent on hERG datasets by different methods

To evaluate the efficiency of the seven automatic substructure extraction tools, we used a hERG dataset containing 20,000 compounds for testing in this part. First, we evenly sampled data sets of different sizes in the original data set, then used 7 methods to extract substructures under default parameters and recorded the time consumed by each process. The configuration of the computer used in this part was as follows: The CPU was Intel Core i7-10700, the memory was 16 GB, and the memory frequency was 2993 MHz. Figure 6 shows the time consumed by different methods on hERG datasets of different sizes. From this figure, we can see that MoSS mined substructures the fastest, processing 15,000 compounds in about 6 s. This is followed by PySmash_group and PySmash_circular, which both took less than 30 s. The processing speed of SARpy was moderate, and the processing time can be controlled in less than 5 min when processing 15,000 molecules. Neither PySmash_path nor KRFP had the advantages of other methods, and Bioalerts was the slowest, especially when dealing with more than 5,000 compounds. However, in general, the work efficiency of all these methods is acceptable, and the time consumed by each method is tolerable when extracting substructures from small and medium data sets.

### The effect of SAs on QSAR predictive models

**Fig. 7:**
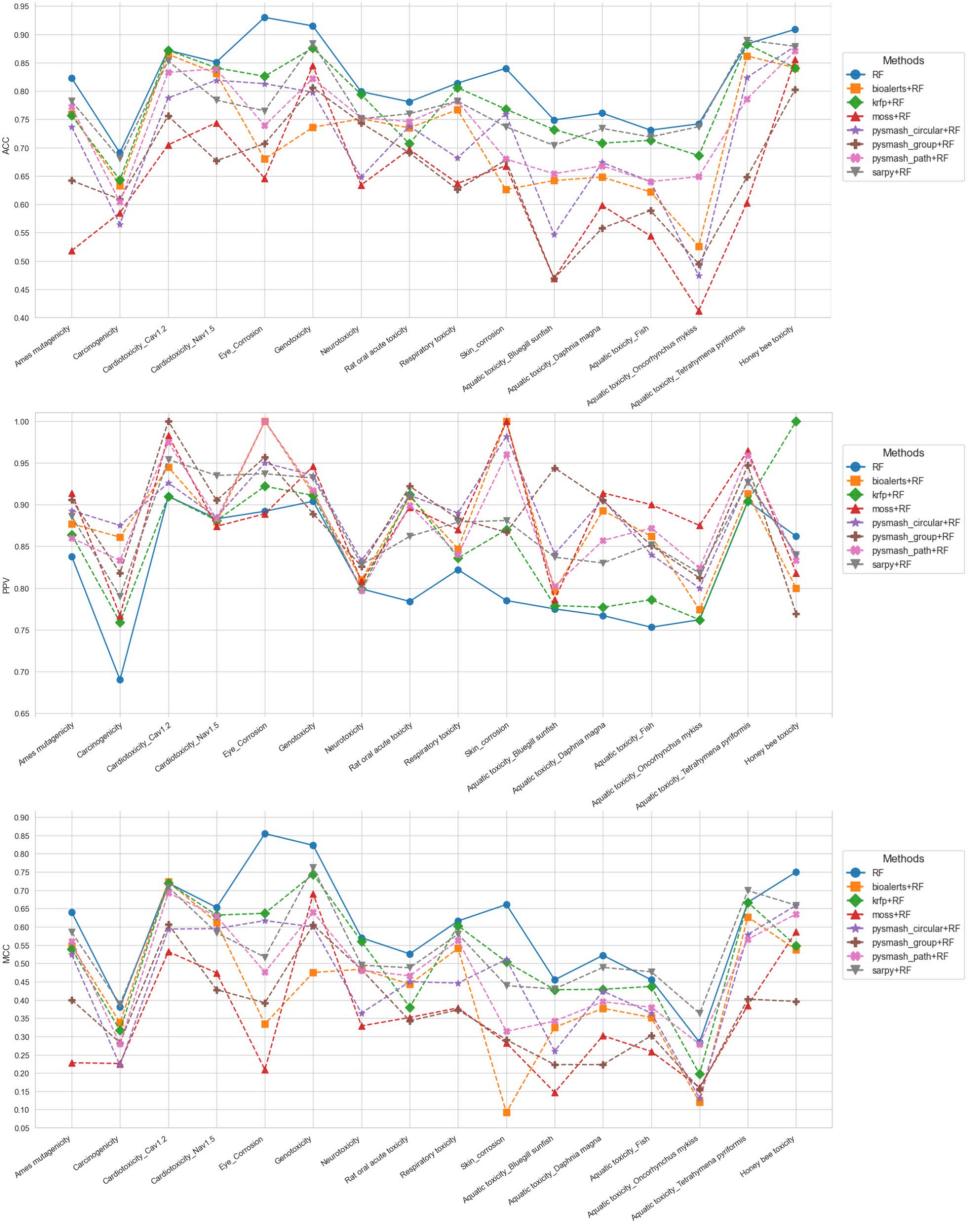
The predictive abilities of the RF and the integrated models on the test sets of 16 toxicity endpoints

Recently, several toxicity prediction studies have shown the benefit of combining mechanism-based alerts with QSAR predictions, enabling both transparency and expert evaluation [1, 35]. Although QSAR modeling is often referred to as a "black box" approach, these models can be interpreted in terms of structural features responsible for activity or toxicity [36–41]. To explore the specific effect of SAs on the prediction of toxicity by QSAR methods, we were encouraged to conduct a detailed comparative analysis in this section. As mentioned in Sect. 3.2, the comparison in this part still focused on the seven methods and the 16 toxicity endpoints that can successfully obtain their substructures. Firstly, each toxicity data set was randomly divided into a training set and a test set with a ratio of 8:2, where the training set is used for model building and the test set is used for the evaluation of QSAR model and substructure-based models. Then, the ECFP4 fingerprint was applied to represent chemical compounds and random forest (RF) was used for model construction [42–44]. Finally, the predictive results of individual QSAR models, individual substructure-based models and integrated models were collated and collected for further analysis. In the integrated models, only compounds with both high predictive score (> 0.5) and the existence of representative substructures will be recognized as positive. Here, the predictive score refers to the probability that the prediction model assigns to a compound being a toxic compound, ranging from 0 to 1. The higher the value, the more likely the compound is to be a toxic one. Figure 7 shows the predictive capabilities of the RF models and the integrated models on the test sets of 16 toxicity endpoints. As can be seen from the figure, from the perspective of ACC and MCC values, almost all RF models outperformed the integrated model. This indicates that the addition of substructures not only fails to further improve the overall accuracy of the QSAR prediction model, but instead leads to a decrease in its predictive ability. However, it is worth noting that, based on the PPV values, all the integration models for 11 out of the 16 toxicity endpoints were significantly superior to the RF models. This confirms that the substructures obtained by all 7 methods can enhance the recognition ability of the QSAR models for toxic compounds and make them interpretable. In certain scenarios, such as when screening the toxicity of compounds with low QSAR scores, the presence of toxic substructures plays a decisive role. Without the indication and interpretability of representative substructures, these compounds with definite toxicity might be considered for further development, thereby causing incalculable economic losses and safety hazards. In conclusion, based on the comparison results of this section, we can draw a conclusion that compared with the individual QSAR models, the integrated models incorporating substructures have greater advantages in identifying toxic compounds, compensating for the lack of interpretability of black-box models, and providing a more comprehensive perspective for the early screening of toxic compounds.

## Conclusion

In recent years, a large number of rigorous studies have shown that structural alerts have good visualization and interpretability, and play an important role in drug development, especially in early toxicity assessment. Presently, many automatic substructure extraction tools with different characteristics and extraction capabilities have emerged to meet the needs of drug developers to obtain toxic substructures efficiently. To evaluate different types of substructure extraction tools and identify the optimal one, we conducted a comprehensive evaluation of 7 representative popular tools based on 43 toxicity datasets, including the tolerance of the method, the number of substructures, the carrying information of substructures, the reliability of substructures, the predictive ability of the substructure model, and the processing efficiency of different methods. The results showed that PySmash_circular had the best performance overall, with reasonable data tolerance, suitable numbers of substructures, acceptable work efficiency, satisfactory substructure indicators, and excellent global predictive ability. SARpy performed best in the evaluation of substructure models, but it focused more on PR values of detected SAs. PySmash_path and Bioalerts performed well in all respects, but they required more uptime and provided users with too many substructures. PySmash_group had the best data tolerance, but its predictive power was not as good as the other two PySmash approaches. MoSS was the most efficient, but neither it nor KRFP performed as well as the other five methods throughout the evaluation. The comparative study with the RF model also found that the substructures detected by all 7 tools can enhance the ability of the QSAR model to identify toxic compounds, providing a more comprehensive perspective for the early screening of toxic compounds. At the end of this study, a baseline SA database of 43 toxicity endpoints was made available to the public. We hope that the findings of this paper will provide valuable clues for the extraction and application of SAs and contribute to the development of computational toxicology and environmental assessment in a faster and more reliable direction.

## Supplementary Information

Supplementary material 1.

Supplementary material 2.

Supplementary material 3.

Supplementary material 4.

Supplementary material 5.

## Data Availability

All the data supporting the conclusion of this article are available in Supporting Information. SI_1: All the toxicity endpoints and their detailed information including structural SMILES and original references. SI_2: The distribution of PR, CR, and IG values of substructures of 23 toxicity endpoints. SI_3: The original substructure sets, the benchmark substructure sets, and the results of overlap degree of seven methods. SI_4: The top 10 substructures of *oral acute toxicity *and *Aquatic toxicity_fish* extracted by 7 methods, sorted by IG values, along with the comparison results with ToxAlerts. SI_5: The Figures of PPV and MCC values of the prediction models based on 7 different substructure extraction methods and 16 toxicity datasets.
