# Supplementary material for: A comprehensive evaluation of advanced methods for identifying structural alerts using extensive toxicity data
Source: J Cheminform. 2026 Jan 30;18:27. doi: 10.1186/s13321-026-01157-x (PMC12922411; doi:10.1186/s13321-026-01157-x)

**The distribution of PR, CR, and IG values of substructures of 23 toxicity endpoints**

**1. Ames mutagenicity**


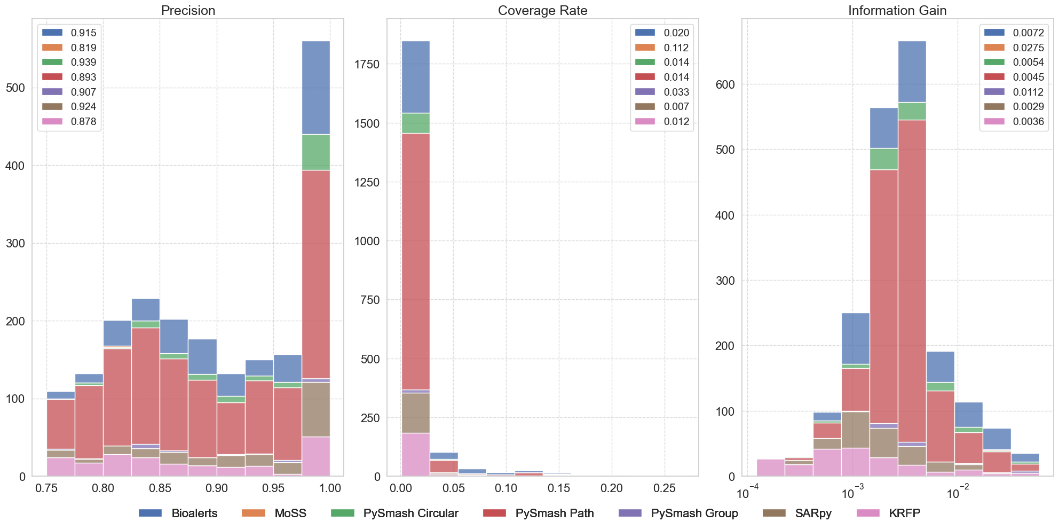


**2. Carcinogenicity**


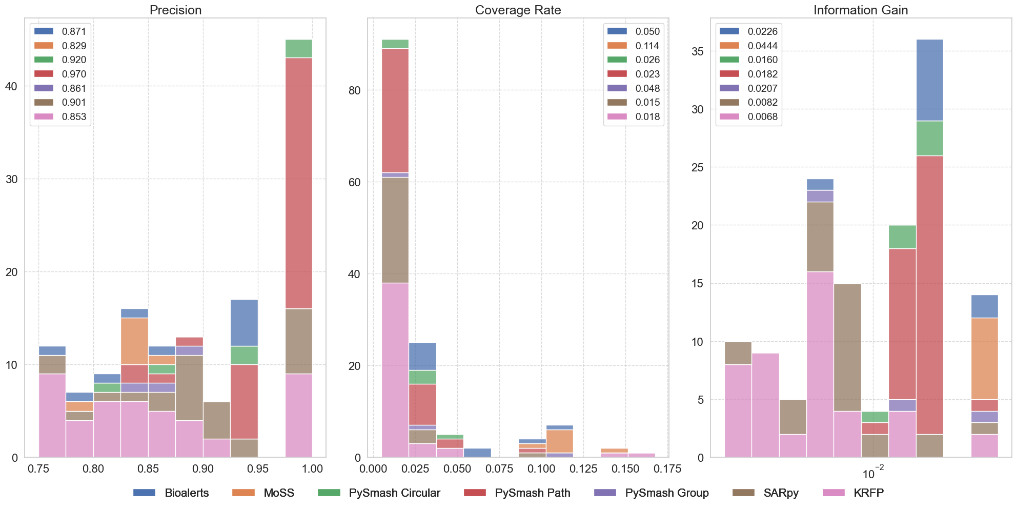


**3. Cardiotoxicity_Cav1.2**


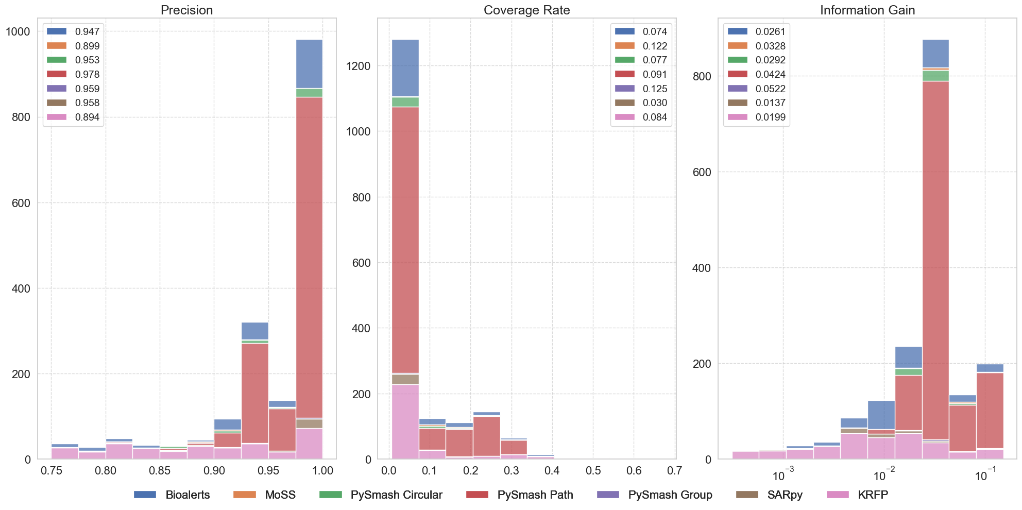


**4. Cardiotoxicity_Nav1.5**


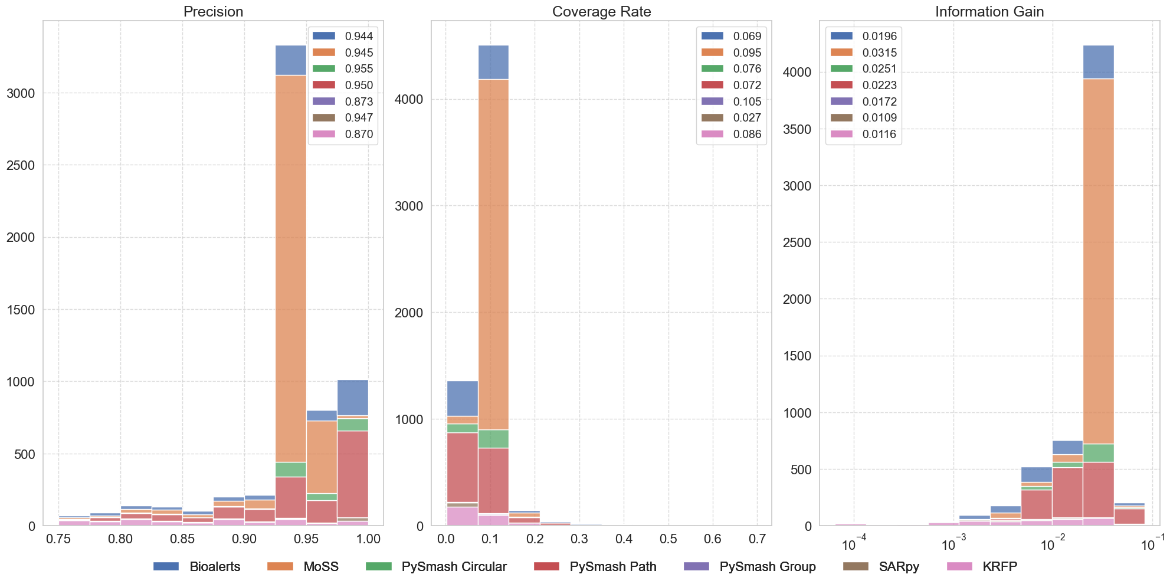


**5. Eye_Corrosion**


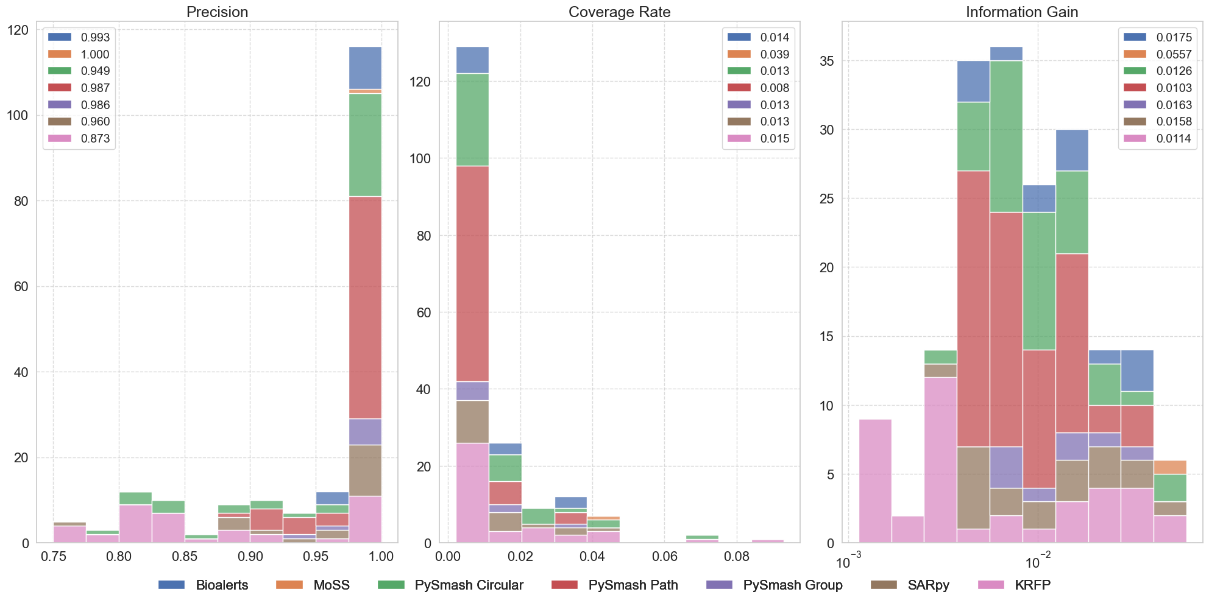


**6. Genotoxicity**


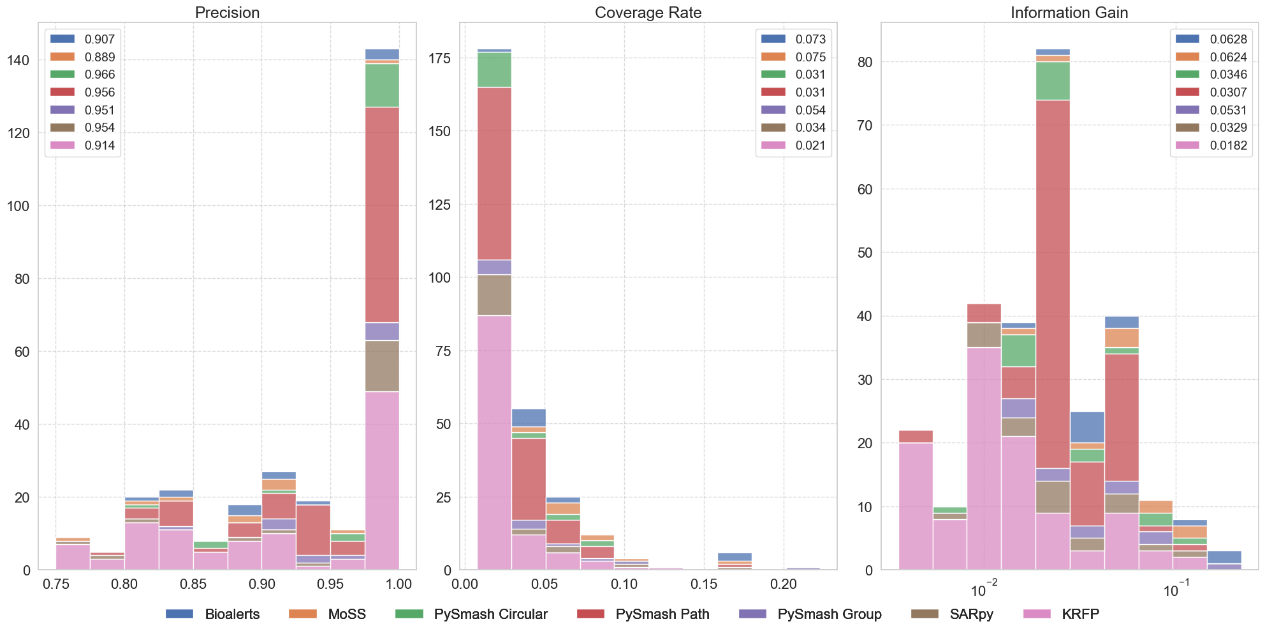


**7. Hemolytic toxicity**


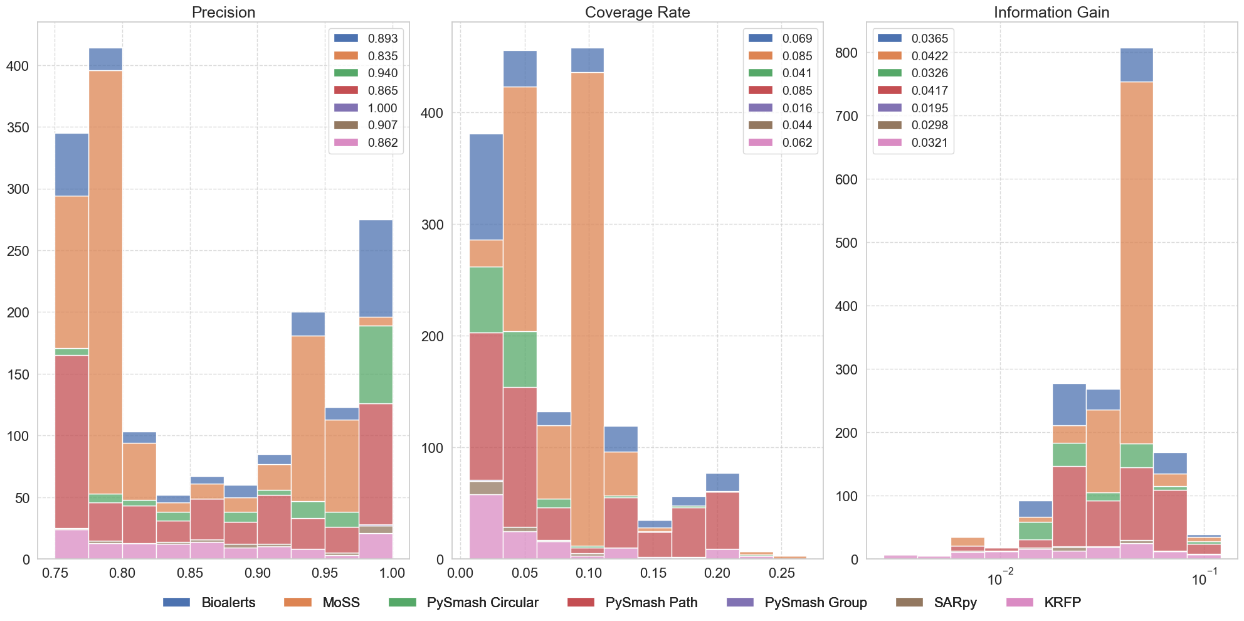


**8. Neurotoxicity**


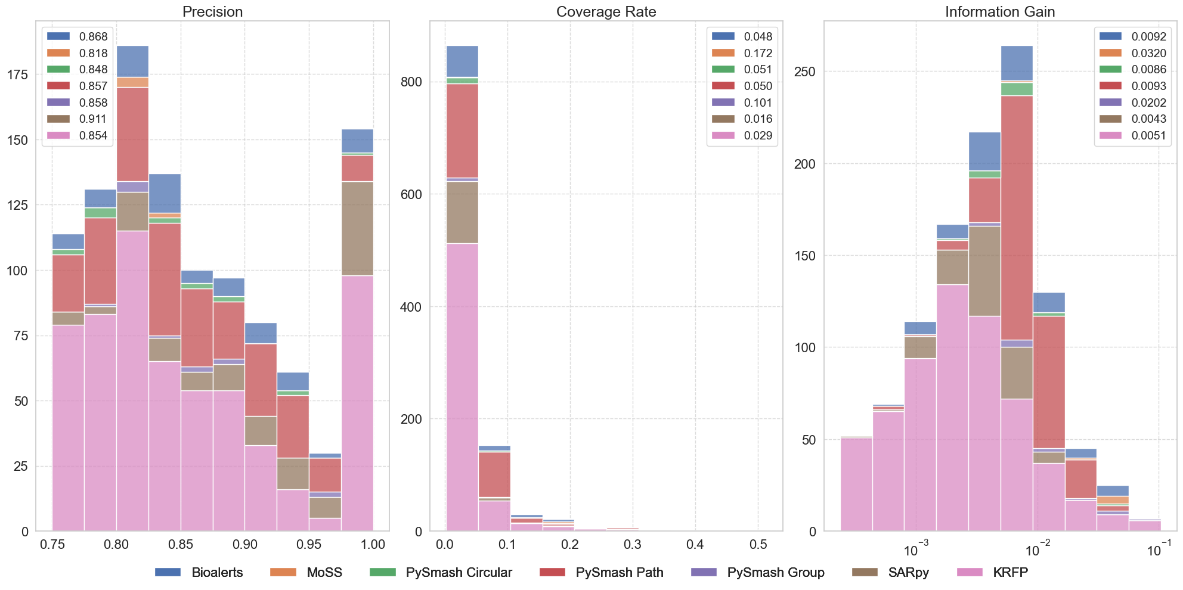


**9. Rat oral acute toxicity**


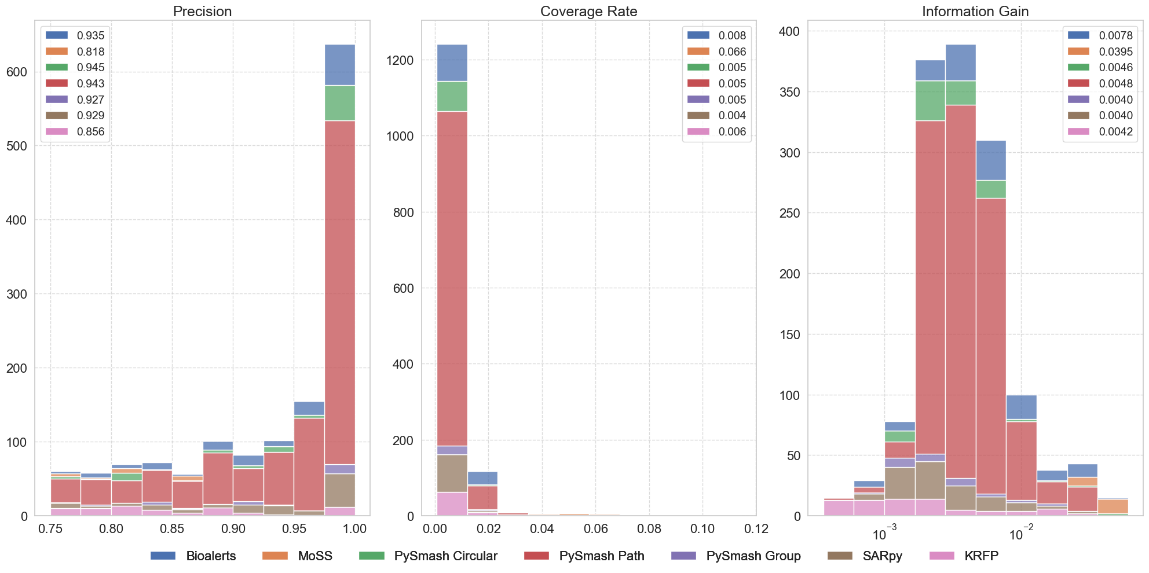


**10. Respiratory toxicity**


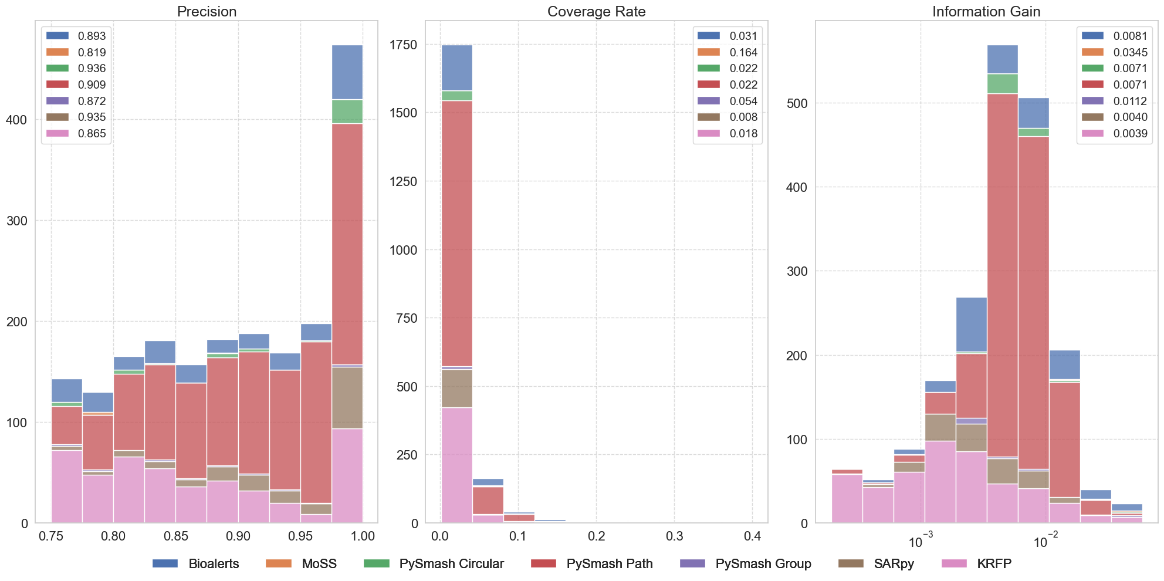


**11. Skin_corrosion**


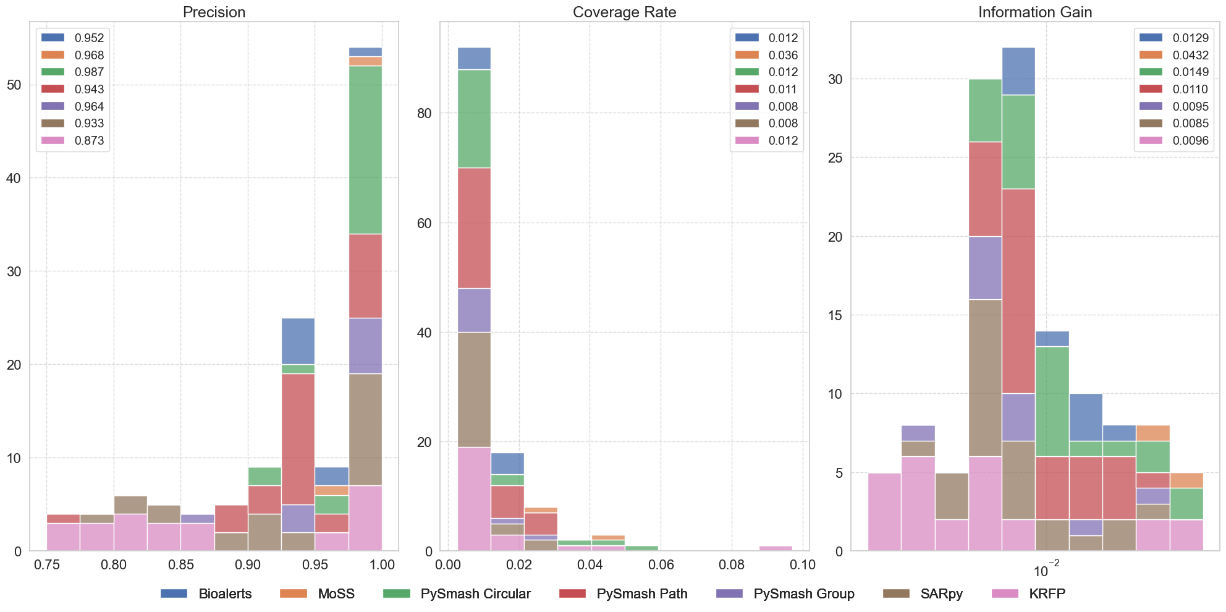


**12. Aquatic toxicity_Bluegill sunfish**


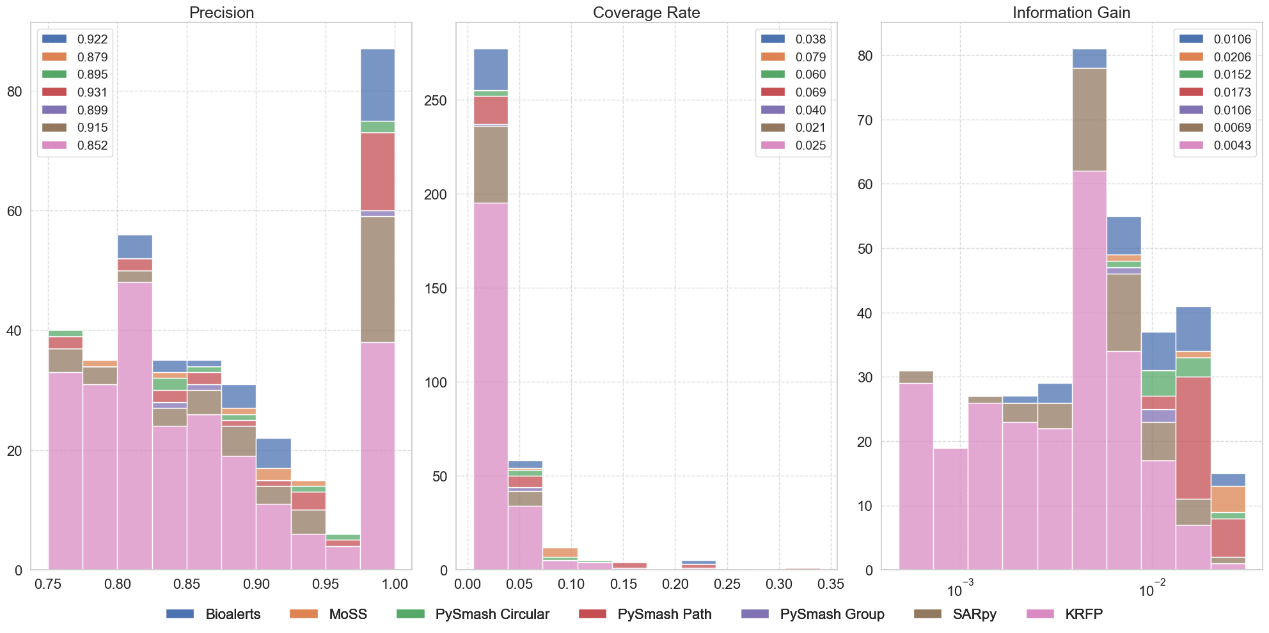


**13. Aquatic toxicity_Daphnia magna**


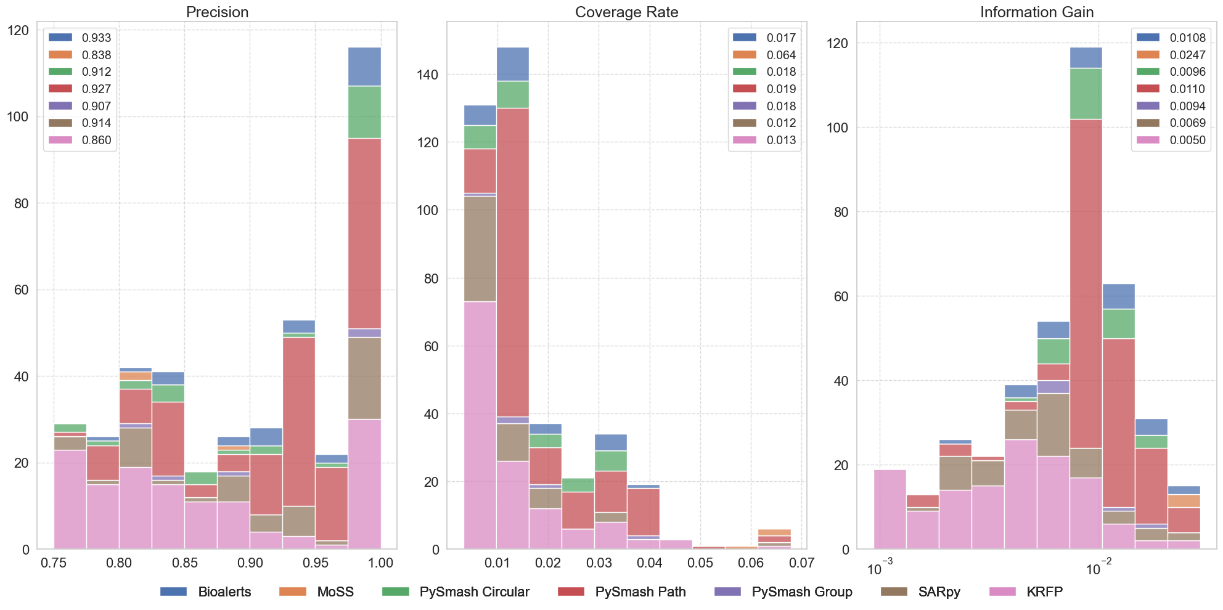


**14. Aquatic toxicity_Fathead minnow**


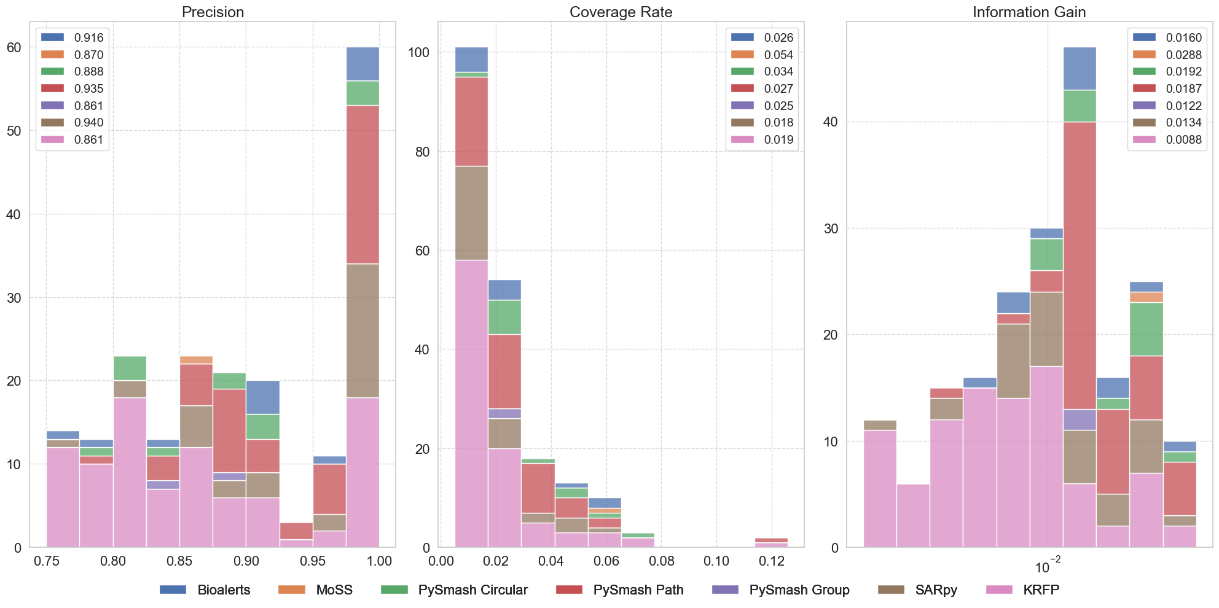


**15. Aquatic toxicity_Fish**


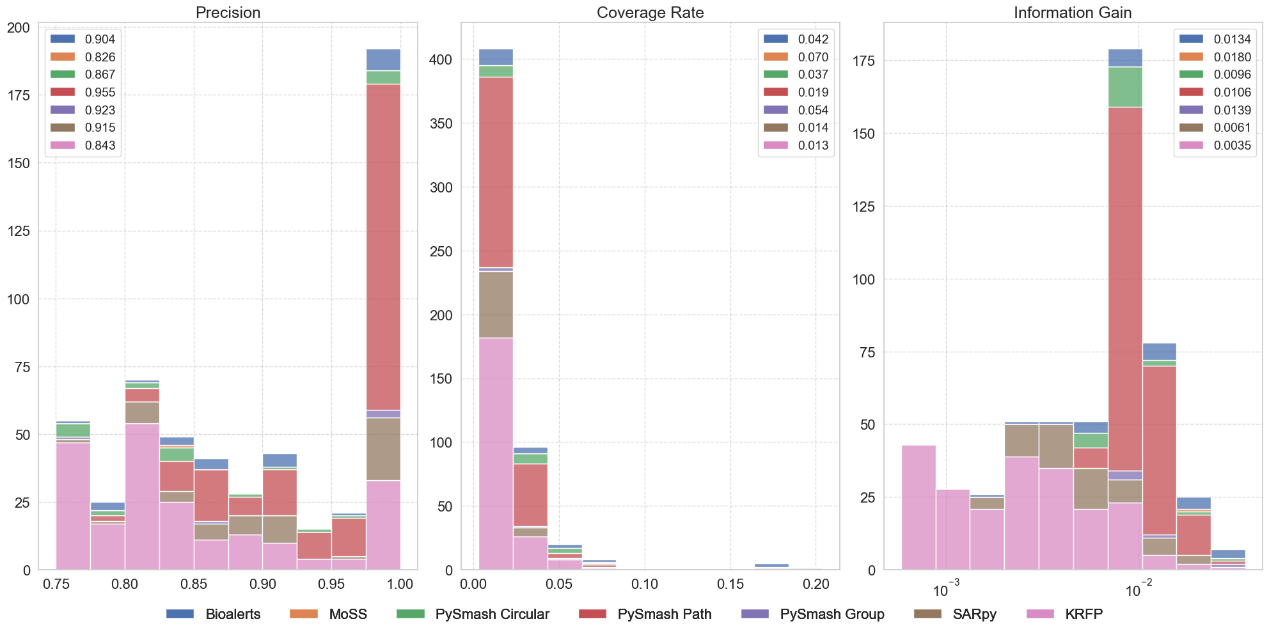


**16. Aquatic toxicity_Oncorhynchus mykiss**


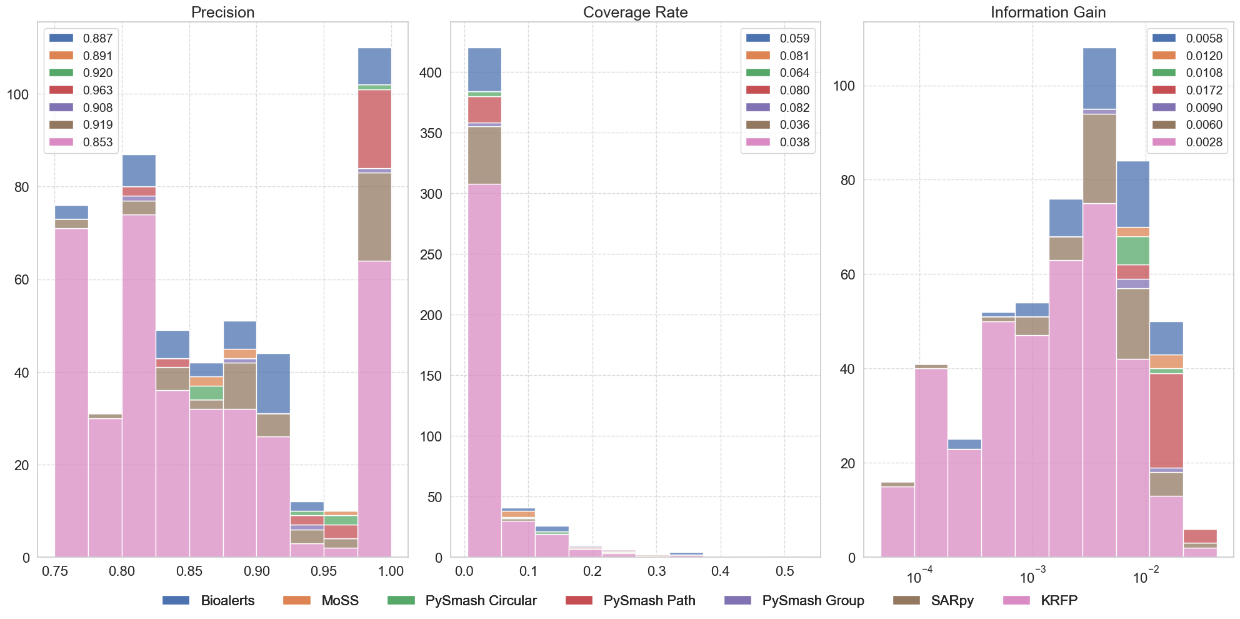


**17. Aquatic toxicity_Pimephales promelas**


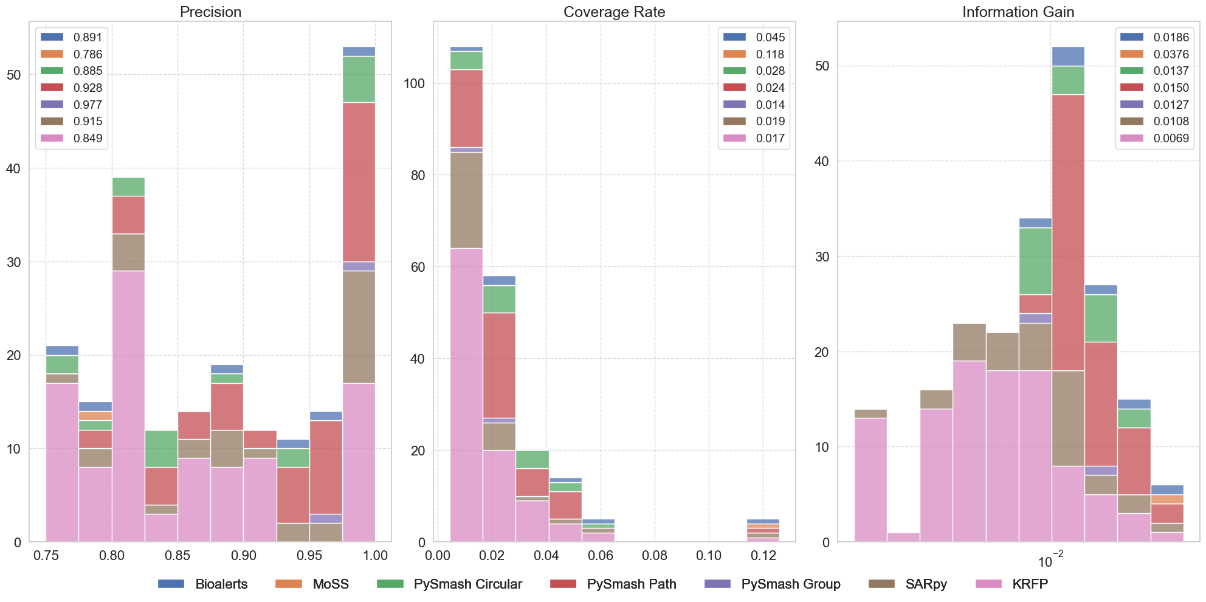


**18. Aquatic toxicity_Pseudokirchneriella subcapitata**


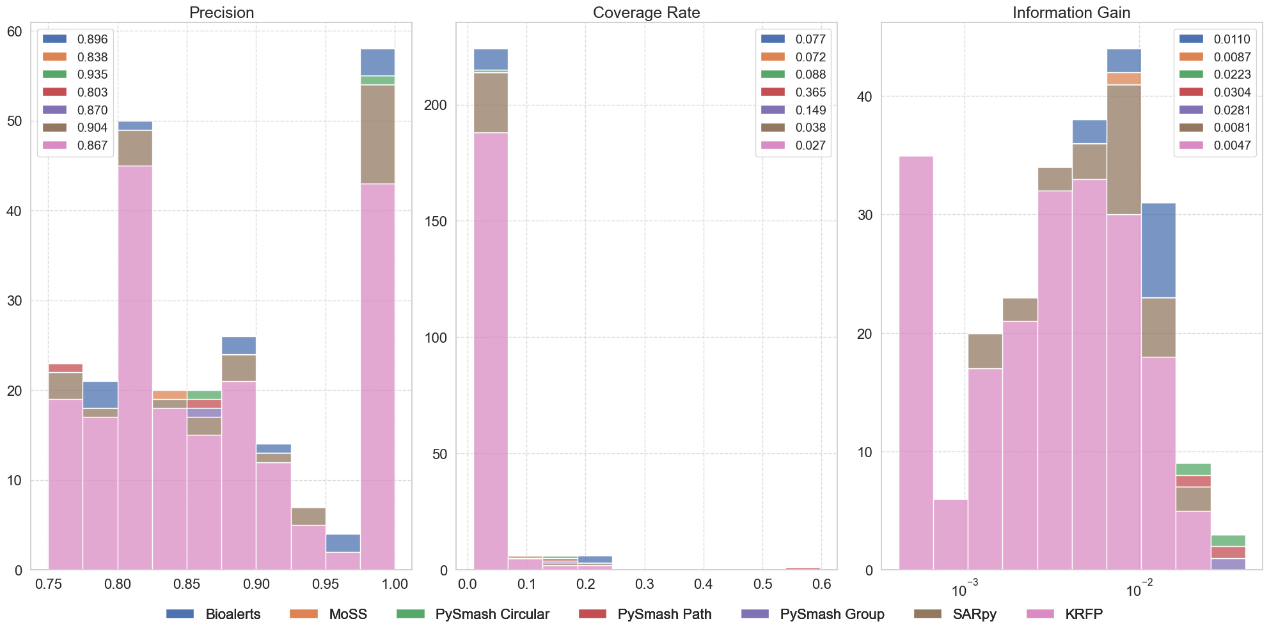


**19. Aquatic toxicity_Sheepshead minnow**


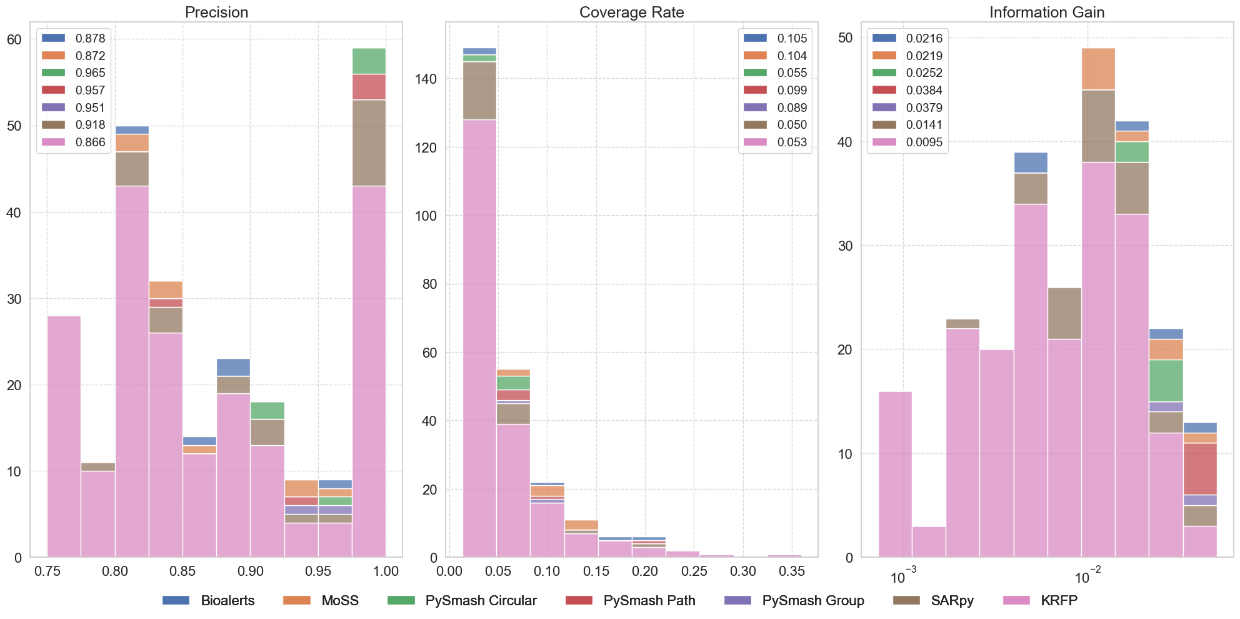


**20. Aquatic toxicity_Tetrahymena pyriformis**


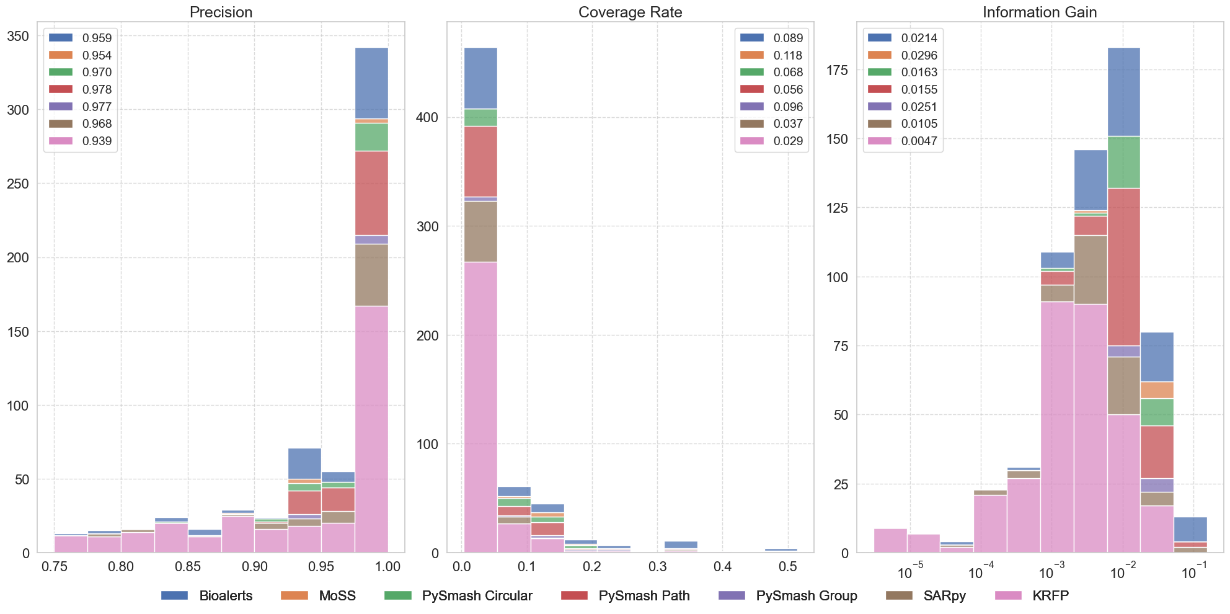


**21. Avian toxicity_mallard duck**


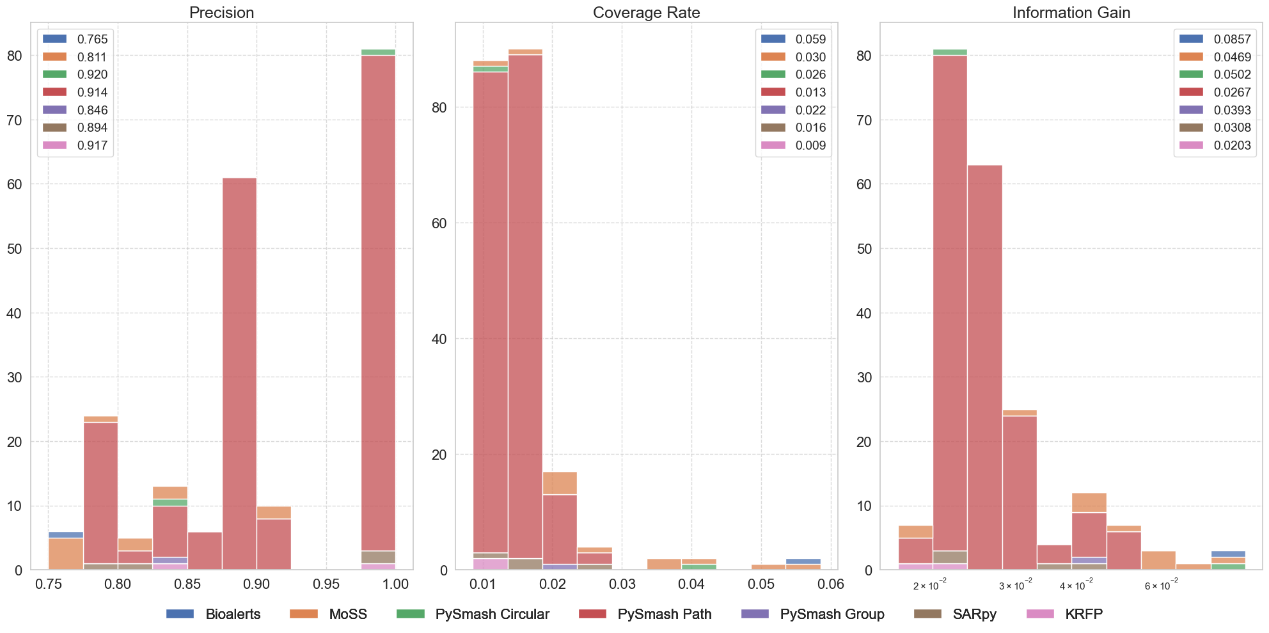


**22. Avian toxicity_northern bobwhite quail**


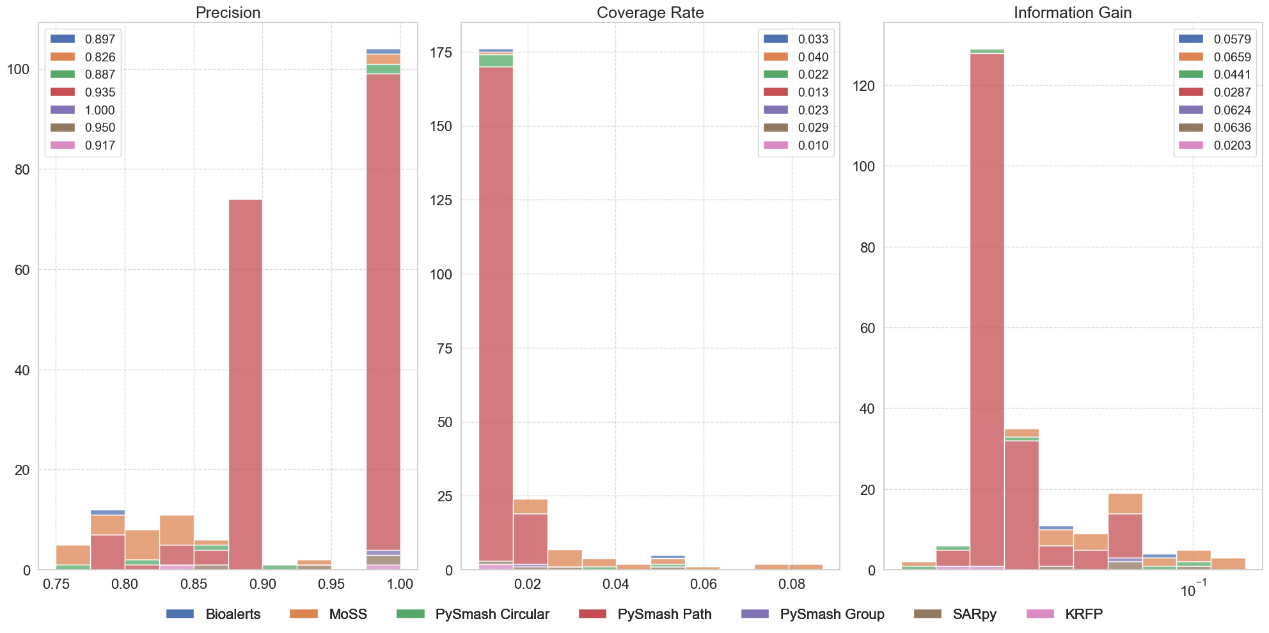


**23. Honey bee toxicity**


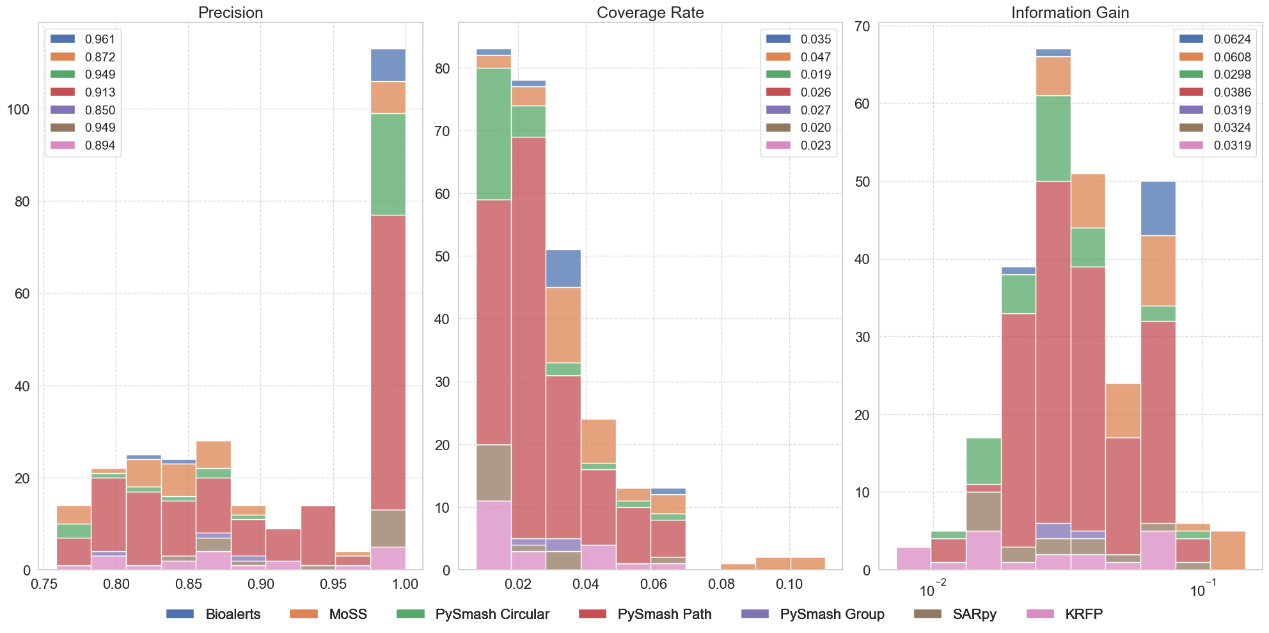

Supplement: Supplementary file 2 — Supplementary material 2. [file 13321_2026_1157_MOESM2_ESM.docx]
