# Supplementary figures and images for: A comprehensive evaluation of advanced methods for identifying structural alerts using extensive toxicity data
Source: J Cheminform. 2026 Jan 30;18:27. doi: 10.1186/s13321-026-01157-x (PMC12922411; doi:10.1186/s13321-026-01157-x)

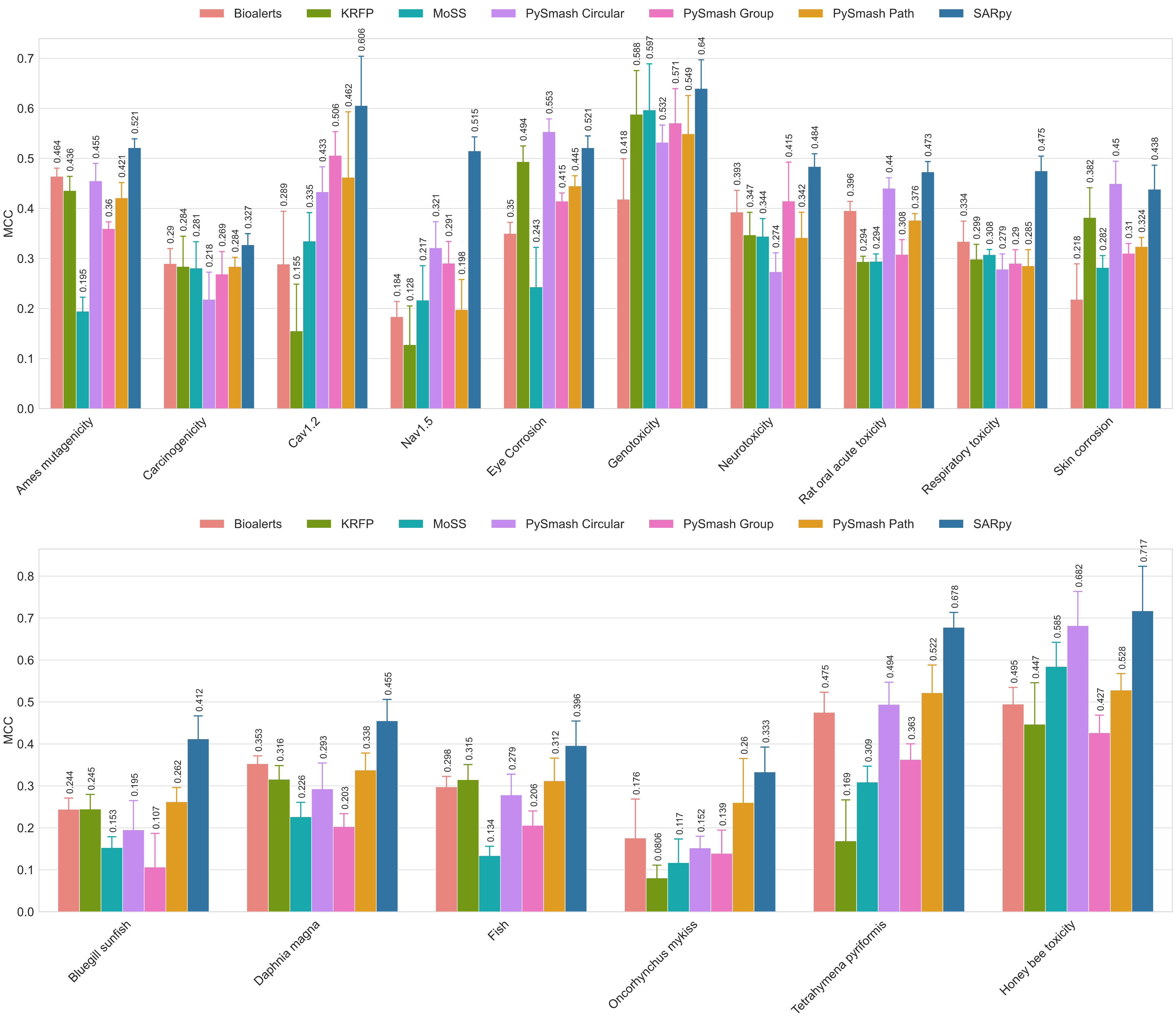

Supplement: Supplementary file 5 — Supplementary material 5. [file 13321_2026_1157_MOESM5_ESM.zip › SI_5_PPV and MCC Figures/test-MCC.jpg]

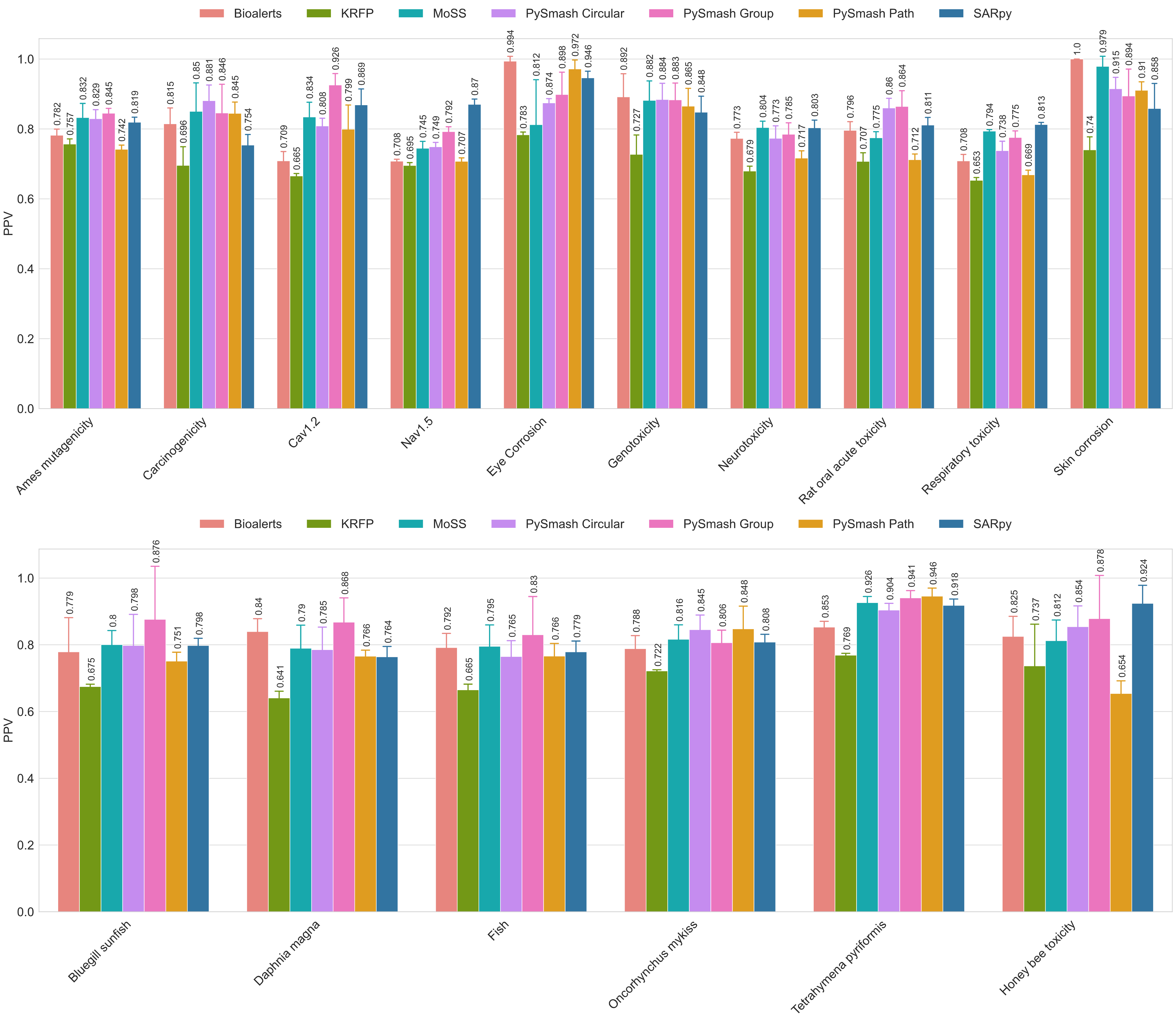

Supplement: Supplementary file 5 — Supplementary material 5. [file 13321_2026_1157_MOESM5_ESM.zip › SI_5_PPV and MCC Figures/test-PPV.jpg]

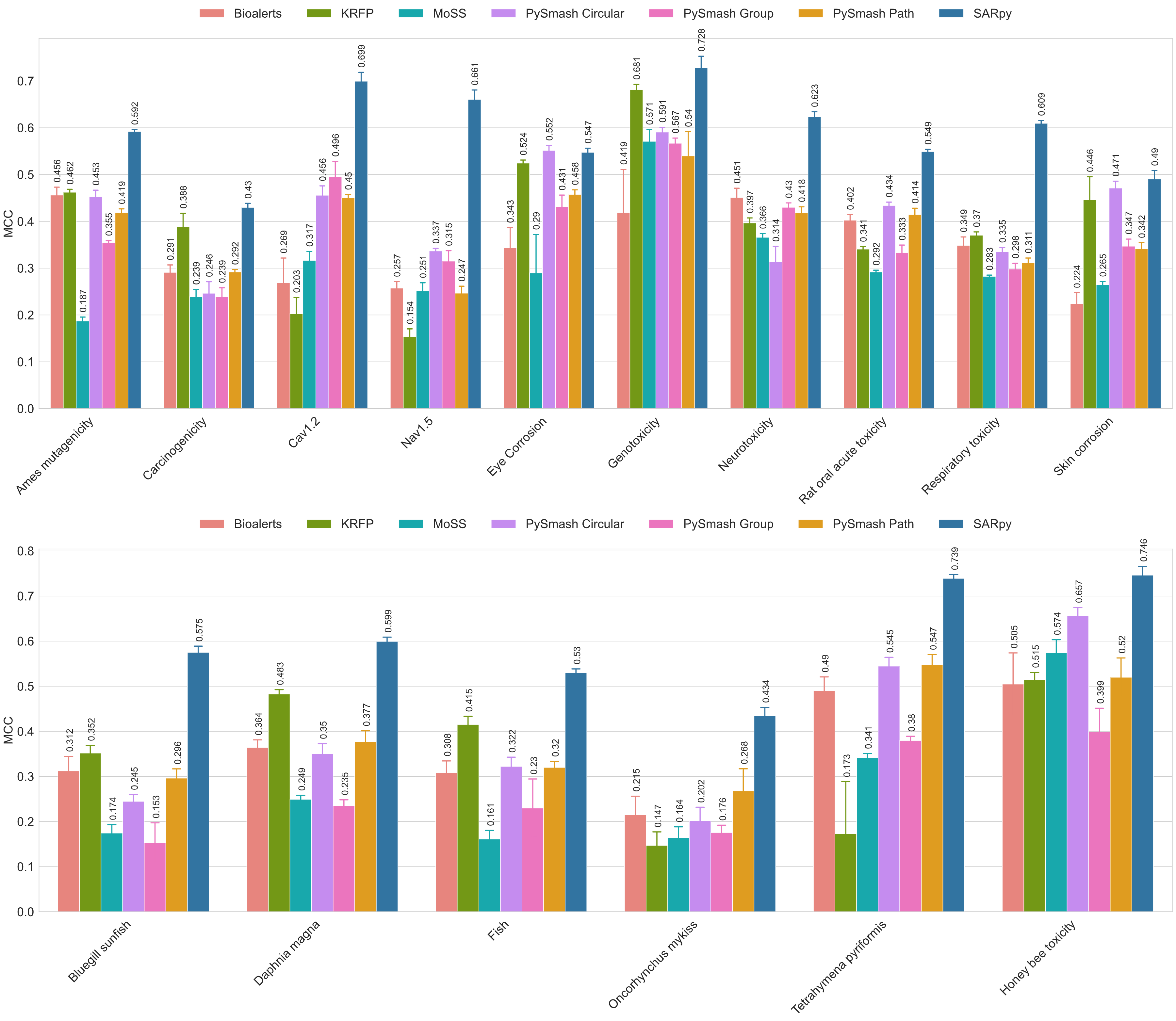

Supplement: Supplementary file 5 — Supplementary material 5. [file 13321_2026_1157_MOESM5_ESM.zip › SI_5_PPV and MCC Figures/training-MCC.jpg]

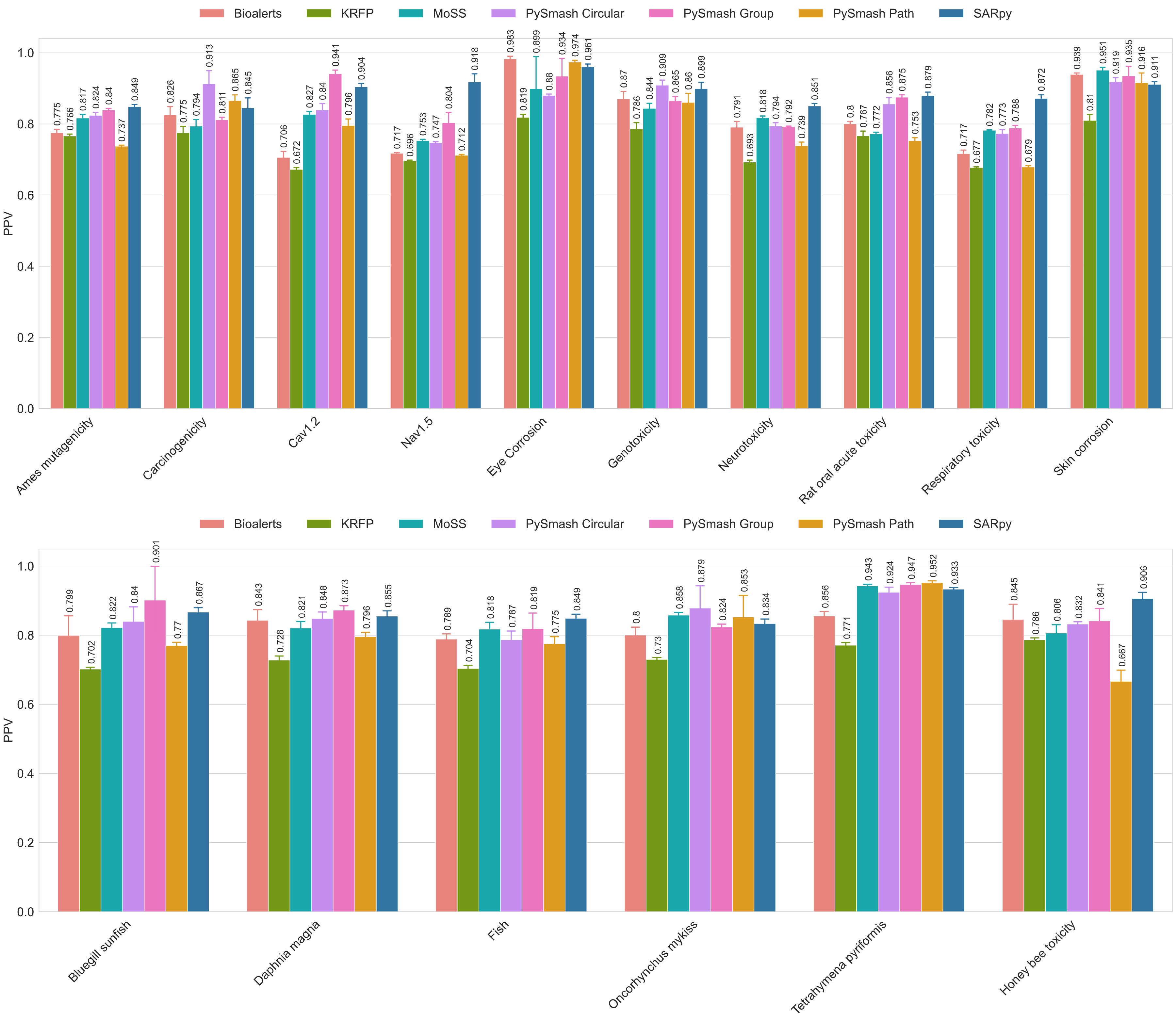

Supplement: Supplementary file 5 — Supplementary material 5. [file 13321_2026_1157_MOESM5_ESM.zip › SI_5_PPV and MCC Figures/training-PPV.jpg]
